# Disentangling the relative roles of resource acquisition and allocation on animal feed efficiency: insights from a dairy cow model

**DOI:** 10.1186/s12711-016-0251-8

**Published:** 2016-09-26

**Authors:** Laurence Puillet, Denis Réale, Nicolas C. Friggens

**Affiliations:** 1 UMR Modélisation Systémique Appliquée aux Ruminants, INRA, AgroParisTech, Université Paris-Saclay, 75005 Paris, France; 2 Département des Sciences Biologiques, Université du Québec à Montréal, Montréal, QC H3C 3P8 Canada

## Abstract

**Background:**

Feed efficiency of farm animals has greatly improved through genetic selection for production. Today, we are faced with the limits of our ability to predict the effect of selection on feed efficiency, partly because the relative importance of the components of this complex phenotype changes across environments. Thus, we developed a dairy cow model that incorporates the dynamic interplay between life functions and evaluated its behaviour with a global sensitivity analysis on two definitions of feed efficiency. A key model feature is to consider feed efficiency as the result of two processes, acquisition and allocation of resources. Acquisition encapsulates intake and digestion, and allocation encapsulates partitioning rules between physiological functions. The model generates genetically-driven trajectories of energy acquisition and allocation, with four genetic-scaling parameters controlling these processes. Model sensitivity to these parameters was assessed with a complete factorial design.

**Results:**

Acquisition and allocation had contrasting effects on feed efficiency (ratio between energy in milk and energy acquired from the environment). When measured over a lactation period, feed efficiency was increased by increasing allocation to lactation. However, at the lifetime level, efficiency was increased by decreasing allocation to growth and increasing lactation acquisition. While there is a strong linear increase in feed efficiency with more allocation to lactation within a lactation cycle, our results suggest that there is an optimal level of allocation to lactation beyond which increasing allocation to lactation negatively affects lifetime feed efficiency.

**Conclusions:**

We developed a model to predict lactation and lifetime feed efficiency and show that breaking-down feed conversion into acquisition and allocation, and introducing genetically-driven trajectories that control these mechanisms, permitted quantification of their relative roles on feed efficiency. The life stage at which feed efficiency is evaluated appears to be a key aspect for selection. In this model, body reserves are also a key component in the prediction of lifetime feed efficiency since they integrate the feedback of acquisition and allocation on survival and reproduction. This modelling approach provided new insights into the processes that underpin lifetime feed efficiency in dairy cows.

**Electronic supplementary material:**

The online version of this article (doi:10.1186/s12711-016-0251-8) contains supplementary material, which is available to authorized users.

## Background

Improving feed efficiency (FE) is a longstanding goal of the livestock sector and is still highly relevant in the current context. Indeed, more efficient animals will produce the same amount of products using less resource and generating less waste in the environment, such as methane or nitrogen. As a result, both pressure on resources (e.g. land use that competes with human food production) and environmental impacts (e.g. greenhouse gas emissions) will decrease. In the past decades, FE of farm animals has increased substantially. For example, Capper et al. [1] reported that, in the USA, the amount of feedstuffs needed to produce one billion kg of milk reached 8.26 × 10^9^ kg in 1944 and only 1.88 × 10^9^ kg in 2007, which corresponds to a 77 % increase in FE. This huge increase in FE was obtained by selecting high-producing genotypes and providing them a high-quality environment to maximize the expression of their production potential. A high level of production leads to a dilution of the fixed costs of production (maintenance requirements and non-productive stages of life) and thus an increase in FE. However, there is growing evidence that this means of increasing FE is not sustainable, particularly for dairy cattle females. The first reason is that a high level of production is negatively associated with other dairy female traits, such as fertility and health [2, 3]. Selection for high production has led to undesired responses by indirect selection that result in greater negative energy balance, i.e. greater body reserve mobilization during early lactation that leads to more reproductive or health problems. As a result, the expected dilution effect linked to higher production may be offset by a decline in productive lifespan because of poor health and/or fertility. If one considers the non-productive period (the phase prior to first calving) of the cow’s life as an efficiency cost to be diluted by the productive part of the cow’s lifespan, then it is clear that reducing the productive lifespan of the cow will decrease lifetime FE. Even if the integration of functional traits into selection indices has, to some extent, limited these negative associations [4, 5], it is far from clear what is the optimal pattern of body reserve usage across the lactation cycle to maximize lifetime FE [3]. A second reason that limits our capacity to sustainably improve FE relates to the role of genotype-by-environment (G × E) interactions on FE and its component traits. The environment in which production occurs will change in the future and breeding objectives will have to account for such changes (for instance, performance under low levels of nutrition or heat stress conditions [6]). In the context of genetic selection for feed efficiency in a future changing environment, we need to know how the environment in which selection is performed shapes the genetic correlations between the component traits of FE. For instance, a strong genetic propensity to accumulate body reserves prior to calving may be negatively correlated with FE in rich environments (where those reserves are less needed), but the converse may be expected in poor or variable environments. These G × E interactions still need to be better experimentally quantified in dairy cows, which until now have been kept in relatively controlled environments, although there is a considerable amount of data for other mammalian species (e.g. rabbits [7], mice [8], and pigs [9]).

Simulations can be a useful tool to explore such contrasting scenarios, provided that the design of the animal “building block” at the heart of the simulation is an appropriate representation of the main biological processes that contribute to, in this case, FE. In animal nutrition, FE is generally considered as the product of digestive efficiency and metabolic efficiency. Digestive efficiency reflects the animal’s ability to acquire nutrients, i.e. intake and digestion, while metabolic efficiency reflects nutrient partitioning and utilization for physiological functions. These two steps in the conversion process can be broadly designated as resource acquisition and allocation. They are both affected by genetic variation and thus contribute to variation in FE [10]. However, in the relatively few nutritional models that include animal genotype, the genotype is invariably included via the concept of production potential, i.e. the maximum amount of a product such as milk that the animal can produce. This is typically used to estimate nutrient requirements and thereby the required diet composition for a given intake level. Given that the total production produced is the product of nutrient intake and nutrient partition, this way of representing the animals’ genotype does not allow the study of the genetic variation in acquisition and in allocation, separately. Thus, to improve our ability to predict the effect of selection on different components of FE, we need to develop simulation models that account for genetic and environmental effects at both the level of acquisition and the level of allocation.

Thus, we developed a mathematical description of the interplay between the main life functions of a dairy cow. This systemic model explicitly integrates energy acquisition and allocation as processes that drive the expression of phenotypic traits, and therefore FE. The model accounts for genetic components in both processes and therefore allows the simulation of genotypes that result from different combinations of acquisition and allocation trajectories.

The aim of this paper is to present the basic assumptions, ideas and design of the model, and the evaluation of its behaviour to variation in four key parameters related to acquisition and allocation. Simulations were used to quantify how changes in parameters that drive acquisition and allocation affect the different definitions of efficiency, thus providing proof-of-concept of the importance of breaking-down FE into these components.

## Methods

### Model description

The model description follows the overview, design concepts, and details (ODD) protocol for describing individual- and agent-based models [11]. The model is currently implemented with Modelmaker version 3.0 (Cherwell Scientific Ltd, 2000).

#### Overview

In order to design a model that represents the animal building block for predicting G × E interactions on feed efficiency (FE), we chose to break the overall process of resource conversion down into three elementary processes: resource acquisition, allocation and utilization.

Resource acquisition is ultimately defined as the input of energy in the organism, resulting from the intake of dry matter (DM) from the environment and its conversion into metabolizable energy (ME) through digestion. Acquisition depends on resource availability (environmental component) and on genetic capacity to acquire resource (animal component). Resource allocation is defined as the partitioning of ME among the following physiological functions: growth, gestation, lactation, maintenance and reserves. Allocation depends on a genetic component and on changes in physiological states. Finally, resource utilization is defined as the conversion of quantities of energy allocated to physiological functions into phenotypes (body mass components, milk, conception and survival probabilities). With this structure based on a decomposition of the processes that generate phenotypes, the model is flexible enough to represent responses to resource availability through variation of acquisition, variation of allocation or a combination of both.

As proposed by [12], we consider that gene regulations give rise to meta-mechanisms at the animal level, which can be represented by a set of parameters in a dynamic model of life functions. Our model is based on this principle. It was not designed to capture all the physiological mechanisms that underpin life functions. We consider the dairy cattle female as an active biological entity with its own agenda [13], rather than being a passive convertor of resource into products. This view reflects the fact that gene expression changes with age and physiological state, and thereby the relative priorities among life functions change throughout the female lifespan. For example, cows in early lactation partition energy towards the mammary gland and mobilize body reserves, irrespective of the quality of the feed available. As lactation progresses, cows increasingly partition energy away from milk towards body reserves. These changes in priorities reflect temporal differences in gene expression through the life of the animal that are the result of evolution and that have been further shaped by selection. To capture these changes, genetically-driven lifetime trajectories of acquisition and allocation (DM intake and energy partition) are assumed. They provide the dynamics that control the flow of resources to different life functions; the efficiency of utilization of these resources is assumed not to change with time and physiological state. Both resource acquisition and resource allocation trajectories can be modulated via genetic-scaling parameters, which allow the representation of the between-animal innate variability in these processes. These are not breeding values per se, rather they are multipliers on acquisition and allocation trajectories and thus provide the means to represent differences between genotypes in acquisition and allocation, as proposed by [12]. For acquisition, the genetic-scaling parameters operate on the maximum intake reached at maturity and during lactation. For allocation, the genetic-scaling parameters operate on the rate of transfer of priorities between life functions. In our study, the model represents a single cow with genetic-scaling parameters as independent inputs that reflect its genotype. We do not consider that the model represents the mean of a population but rather, that it provides the elementary animal unit for building virtual populations in an individual-based population model to study the effects of selection. In this context, it will be possible to set different heritability values and different genetic correlations between the parameters of the model to study how genetic constraints will affect the evolution of the cow’s FE. On the basis of acquisition and allocation trajectories that are driven by genetic-scaling parameters, and resource availability, the model simulates trajectories of phenotypes (DM intake, quantities of energy, body mass components and milk production) and timings of reproductive events throughout the lifespan of an individual cow. With this representation of the animal, the phenotypic expression of a genotype permitted by the environment can be simulated during different phases over which FE is determined and different sources of variability in FE can be better decomposed.

#### Design and concepts

**Fig. 1:**
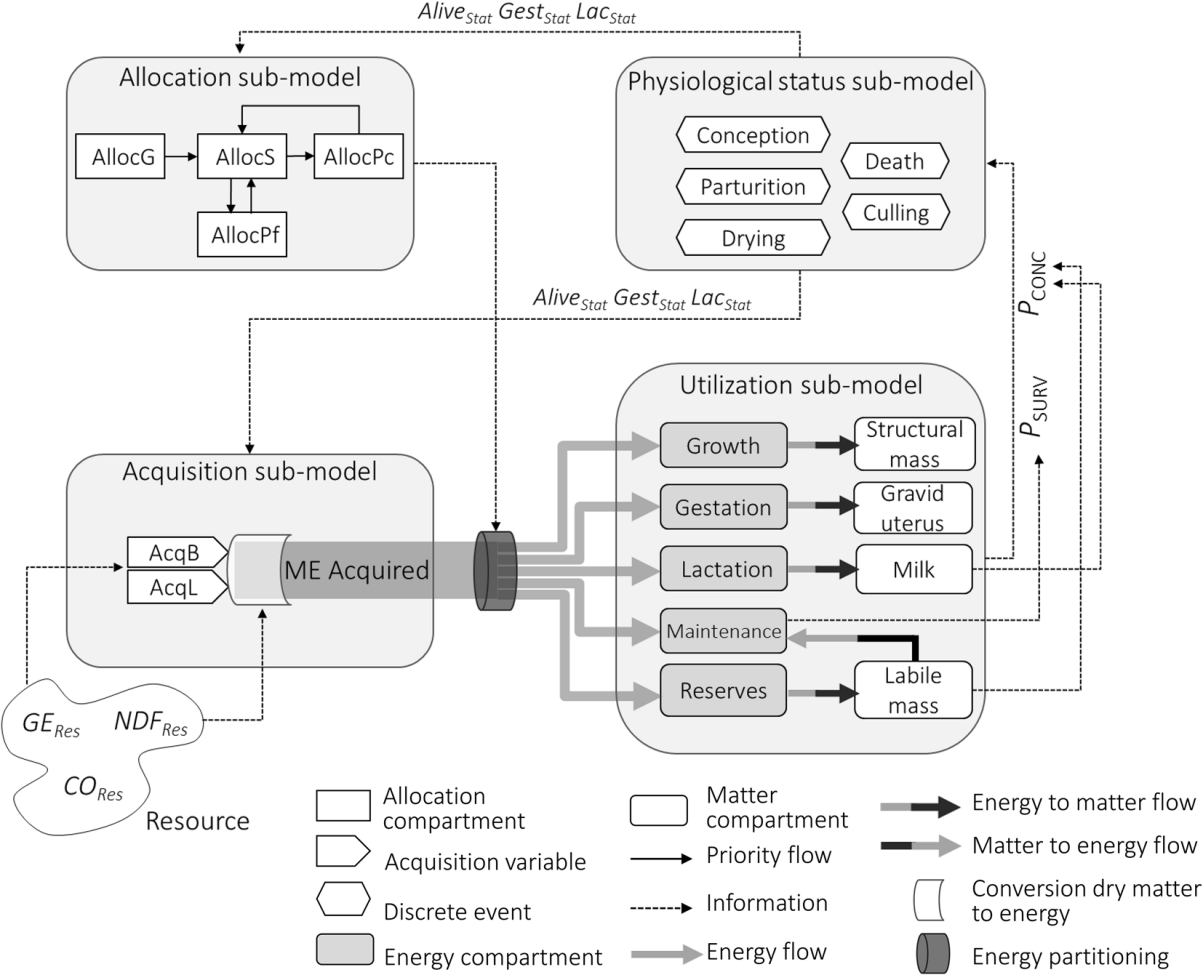
Conceptual diagram of the model illustrating the connections among sub-models. *AllocG*, allocation to growth; *AllocS*, allocation to survival; *AllocPf*, allocation to future progeny; *AllocPc*, allocation to current progeny; *AcqB*, basal acquisition; *AcqL*, lactation acquisition; *ME Acquired*, metabolizable energy acquired; *GE*_*Res*_, resource gross energy density; *NDF*_*Res*_, resource fiber content; *CO*_*Res*_, proportion of concentrate feedstuff in resource; *P*_*SURV*_, probability of survival; *P*_*CONC*_, probability of conception; *Alive*_*Stat*_, Boolean for living status; *Gest*_*Stat*_, Boolean for gestating status; *Lac*_*Stat*_, Boolean for lactating status

The model structure is made up of four sub-models: acquisition, allocation, utilization and physiological status (Fig. 1). Acquisition and allocation sub-models are core modules that integrate the genetic determinants and lifetime dynamic changes. Utilization and physiological status are supporting modules that are based on simple principles and existing approaches. They are not the focus of the modelling effort since our aim is not to study the mechanisms that are associated with energy utilization and reproduction.

**Fig. 2:**
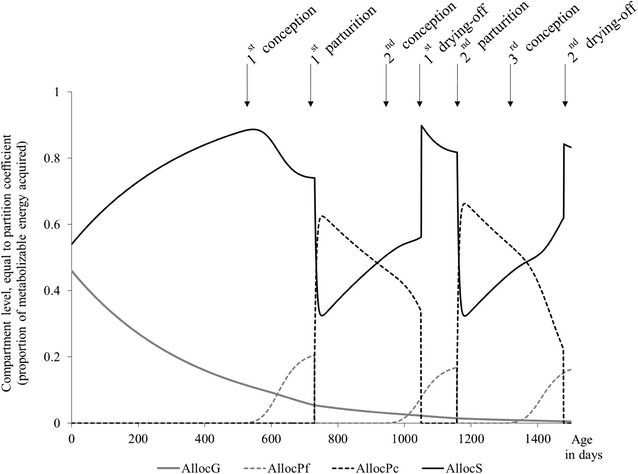
Dynamic changes in the allocation sub-model over two reproductive cycles of a dairy cow. *AllocG*, allocation to growth; *AllocPf*, allocation to future progeny; *AllocPc*, allocation to current progeny; *AllocS*, allocation to survival

The allocation sub-model is the core of the model and drives the partitioning of ME between physiological functions. It accounts for changes in priorities during the lifespan of the animal by generating genetically-driven dynamic changes in coefficients of partition among four life functions: growth, future progeny, current progeny and survival. As proposed by [14], genetically-driven changes refer to any change that occurs in cows kept in a non-constraining environment. Dynamic changes of these compartments are illustrated in Fig. 2.

The compartment *AllocG* represents the female priority for growth. Its level gives the coefficient of partition for growth function, i.e. the proportion of acquired energy that is allocated to growth. The compartment *AllocPf* represents the female priority for its future progeny. Its level gives the coefficient of partition for the gestation function. The compartment *AllocPc* represents the female priority for its current progeny. Its level gives the coefficient of partition for the lactation function. Finally, the compartment *AllocS* represents the priority for survival. Its level gives the coefficient of partition for somatic functions, defined as body mass maintenance and body reserves. Compartments are linked by flows that represent transfers of priorities among life functions. These transfers lead to changes in coefficients of partition (level of compartments) and to a switch in energy investment when the resulting coefficients are used in the utilization sub-model. A dimensionless quantity of one is moving in the network of compartments to represent transfers of priorities. Therefore, by construction, the sum of the partitioning coefficients is equal to 1. This ensures a neutral balance between energy acquired and energy allocated to functions. During early life, the female’s priority for growing progressively switches to survival with age. The proportion of energy for growth is high after birth but the priority for growth progressively declines as it approaches maturity by transferring priority towards survival functions with an increasing allocation to the benefit of energy for somatic functions. At first conception, the female’s priority switches from survival to future progeny. An increasing proportion of energy is invested in gestation function, at the expense of the proportion of energy for somatic functions. At parturition, the female’s priority switches from future progeny to current progeny. No more energy is invested for gestation and an increasing proportion of energy is invested for lactation. The female’s priority for its current progeny decreases as lactation progresses, to the benefit of the priority for the female’s own survival. The proportion of energy invested for lactation decreases while the proportion for somatic functions increases. When drying-off occurs, there is a discrete shift in priority between current progeny and survival: energy is no longer invested in lactation. In addition to the control of priority flows by changes in the female’s physiological status (conception and parturition), the priority flows of the allocation sub-model are driven by genetic-scaling parameters. They control priority flows between *AllocG*, *AllocS*, *AllocPf*, and *AllocPc*. Implementation of different values for these parameters corresponds to different rates of priority transfers among functions and this allows the simulation of genetic differences in the profiles of allocation to growth, to gestation and to lactation. The values of these parameters are independent model inputs, therefore, in our study, the genetic differences for each allocation profile are independent of each other.

**Fig. 3:**
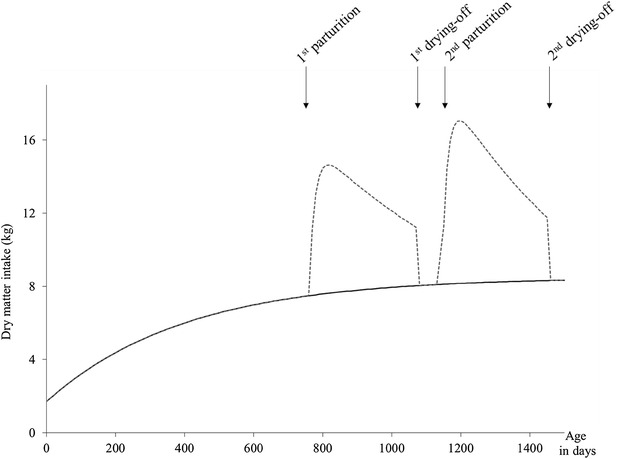
Dynamic changes in dry matter intake in the acquisition sub-model over two reproductive cycles of a dairy cow. Total dry matter intake is made up of a basal component (*AcqB*, *solid line*) and a lactation component (*AcqL*, *dotted line*)

The acquisition sub-model is the second core sub-model since it simulates dynamic changes in dry matter intake throughout lifetime. Acquisition is made up of a basal acquisition component, *AcqB* and a lactation acquisition component, *AcqL* as illustrated on Fig. 3. The basal component describes the maturation of the biological structures linked to resource acquisition as the female matures. The lactation component represents the increase in resource acquisition that is induced by the lactating status. As for allocation, in addition to changes in physiological status, dynamic changes in acquisition are driven by the genetic-scaling parameters, *AcqB*_*GEN*_ and *AcqL*_*GEN*_, allowing the scaling of DM intake curves. Different values can be implemented to simulate genetic differences in the acquisition profiles among individuals.

The dynamic variables that are generated by acquisition and allocation sub-models are combined in the utilization sub-model. This sub-model encodes the conversion of the energy allocated to physiological functions into matter, based on efficiency coefficients and energy contents of this matter. Material variables such as the level of milk production are then used to compute conception probability and survival probability. The physiological status sub-model uses these probabilities to determine the female’s status.

The model uses a time step of 1 day. Simulation starts at birth and stops at the female’s death or culling. At each time step, all the elements are updated simultaneously (Runge–Kutta 4 numerical integration with a fixed time step for compartments). Processes that occur within a time step represent daily biotransformation, from acquisition of dry matter to phenotypes. Updated phenotypes are then used by the discrete events of the physiological status sub-model and may lead to a change in the female’s status (gestating, lactating, alive), that is effective at the next time step. In this study, we consider a constant nutritional environment (resource availability, energy density, proportion of fibres and concentrate in the diet) but temporal variation in the environment could easily be implemented.

#### Model details and assumptions

To better understand the dynamic nature of the model, we present the main details of allocation and acquisition and we highlight the linkage, and thus in-built coherence, between life functions. All parameters, compartments, flows and variables are defined in Tables S1 and S2 [see Additional file 1: Table S1 and Additional file 2: Table S2]. Discrete events are described in Table S3 [see Additional file 3: Table S3].

##### Allocation sub-model

**Fig. 4:**
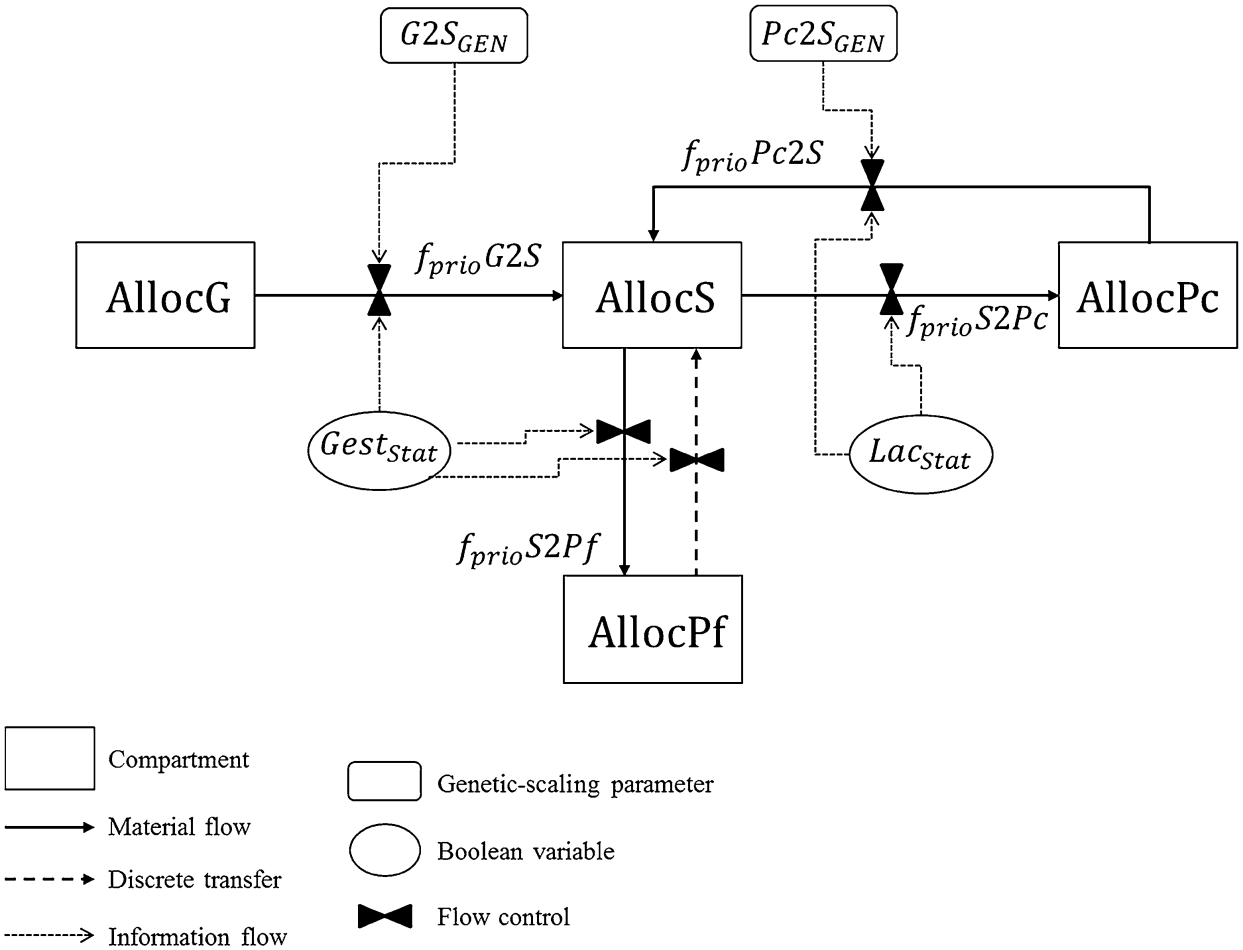
Structure and control of the allocation sub-model. *AllocG*, allocation to growth; *AllocS*, allocation to survival; *AllocPf*, allocation to future progeny; *AllocPc*, allocation to current progeny; *f*_*prio*_ *G*2*S*, priority flow from growth to survival; *f*_*prio*_ *S*2*Pf*, priority flow from survival to future progeny; *f*_*prio*_ *S*2*Pc*, priority flow from survival to current progeny; *f*_*prio*_ *Pc*2*S*, priority flow from current progeny to survival; *Gest*_*Stat*_, Boolean for gestating status; *Lac*_*Stat*_, Boolean for lactating status; *G*2*S*_*GEN*_, genetic-scaling parameter driving allocation to growth by controlling priority transfer from growing to survival; *Pc*2*S*_*GEN*_, genetic-scaling parameter driving allocation to lactation by controlling priority transfer from current progeny to survival

The allocation sub-model is made up of four compartments that reflect the priorities for four life functions: growth, future progeny, current progeny and survival. Dynamics of these compartments are based on mass action laws to represent the progressive transfers of priority among functions across various physiological states. The structure of the allocation sub-model is in Fig. 4, where the amounts of priority for the different life functions are given by *AllocG* for growth (priority for growing), *AllocS* for somatic functions (priority for survival), *AllocPf* for gestation (priority to future offspring), and *AllocPc* for lactation (priority to current offspring).

The transfers of priority are given by the flows *f*_*prio*_ *G*2*S* (priority transfer from growth to survival), *f*_*prio*_ *S*2*Pf* (priority transfer from survival to future progeny), *f*_*prio*_ *S*2*Pc* (priority transfer from survival to current progeny) and *f*_*prio*_ *Pc*2*S* (priority transfer from current progeny to survival). These flows generate the changes in compartment levels and thus the dynamic changes in the allocation of energy to the associated function. They are activated or inactivated depending on the physiological state. They are modulated by the two genetic-scaling parameters. The rate of change in the proportion of energy allocated to growth, *AllocG* is defined by the following differential equation:

1 $$ \frac{dAllocG}{dt} = - f_{prio} G2S. $$

The flow *f*_*prio*_ *G*2*S* represents the decrease in allocation to growth as the female ages. It is given by:

2 $$ f_{prio} G2S = AllocG \cdot G2S_{GEN} + 0.01 \cdot AllocPf, $$

where parameter *G*2*S*_*GEN*_ is the genetic-scaling parameter that defines the rate of priority transfer from growth to survival. An increase in *G*2*S*_*GEN*_ leads to a greater priority transfer and a larger decrease in the *AllocG* level and thus a decrease in the proportion of energy allocated to growth. The priority transfer from growth to survival is further increased by *AllocPf*. Thus, when gestation starts, decrease in allocation to growth is accelerated to enable a greater priority transfer towards future progeny. It is assumed that gestation slows down growth.

The proportion of energy allocated to gestation is given by *AllocPf*, the rate of change of which is defined by the following differential equation:

3 $$ \frac{dAllocPf}{dt} = + f_{prio} S2Pf. $$

The flow *f*_*prio*_ *S*2*Pf* represents the increase of allocation to gestation as gestation time increases. It is modelled with a rising sigmoid function that depends on gestation time and on four fixed parameters (*k*_*H*_ *Pf*_0_, *k*_*H*_ *Pf*_1_, *k*_*H*_ *Pf*_2_ and *k*_*H*_ *Pf*_3_), as we currently assumed no variability for allocation to gestation [see Additional file 1: Table S1]. The proportion of energy allocated to lactation is given by *AllocPc* that is defined by the following differential equation:

4 $$ \frac{dAllocPc}{dt} = \left( { + f_{prio} S2Pc - f_{prio} Pc2S} \right). $$

The increase in the proportion of energy allocated to milk production at the beginning of lactation is described by *f*_*prio*_ *S*2*Pc* as given by:

5 $$ f_{prio} S2Pc = AllocS_{2} \cdot S2P_{c} \cdot Lac\_Stat. $$

The parameter *S*2*P*_*c*_ drives the priority transfer from survival to current progeny at the beginning of lactation. In the current version, it is fixed since no genetic variance in the priority transfer from survival to current progeny is assumed.

The decrease in energy for milk production as lactation progresses is described by the flow *f*_*prio*_ *Pc*2*S*, given by:

6 $$ f_{prio} Pc2S = AllocPc \cdot Lac_{Stat} \cdot \left( {Pc2S_{GEN} + AllocPf \cdot Gest_{Stat} \cdot 0.06} \right). $$

The parameter *Pc*2*S*_*GEN*_ is the genetic component for the priority transfer from current progeny to somatic functions. Changing its value affects the dynamics of *AllocPc* and allows the representation of different strategies of lactation allocation. An increase in *Pc*2*S*_*GEN*_ accelerates the rate of priority transfer from lactation to survival *f*_*prio*_ *Pc*2*S*, and thus, decreases the level of allocation to lactation. This priority flow is also affected by the allocation level to future progeny. As gestation progresses, *AllocPf* increases and accelerates the return of priority from *AllocPc* to *AllocS*. This effect accounts for the depressive effect of gestation on lactation [15]. Finally, the proportion of energy allocated to somatic functions is given by *AllocS* defined by the following differential equation:

7 $$ \frac{dAllocS}{dt} = \left( {f_{prio} G2S - f_{prio} S2Pf - f\_prioS2Pc + f\_prioPc2S} \right). $$

Since there is no loss of the dimensionless quantity of one in the compartment network, at each time step, the sum of the compartment’s levels is equal to 1, thus ensuring a neutral balance between energy acquired and energy allocated to functions.

##### Acquisition sub-model

The acquisition sub-model simulates the quantity of DM acquired from the nutritional environment, depending on resource characteristics (gross energy density, fibre content and proportion of concentrate feedstuff), and its conversion into ME through digestion.

The total daily intake of DM, is given by:

8 $$ AcqT = AcqB + AcqL. $$

The variable *AcqB* is defined by:

9 $$ AcqB = \left( {AcqB_{GEN} - 0.8 \cdot AcqB_{GEN} \cdot e^{{ - k_{{AcqB_{MAT} }} \cdot t}} } \right). $$

The parameter *AcqB*_*GEN*_ is the genetic-scaling parameter that drives basal acquisition. It represents the asymptote of the curve, which corresponds to the maximum intake of DM at maturity for a non-lactating animal. Although intake is frequently expressed as a percentage of body weight, we explicitly chose not to do this. It would create an a priori correlation between allocation to growth and acquisition, and thus, prevent the study of the relative roles of these components on the phenotypic traits and FE. The variable *AcqL* is defined by:

10 $$ AcqL = Lac_{Stat} \cdot AcqL_{Max} \cdot AcqL_{Dyn} . $$

The shape of the curve during lactation is given by the *AcqL*_*Dyn*_ component of Equation E15 in Table S2 [see Additional file 2: Table S2]. The maximum DM that is reached during lactation is given by *AcqL*_*Max*_, which depends on the genetic-scaling parameter *AcqL*_*GEN*_. This latter is the maximum DM reached at maturity to account for the maturation of the potential to acquire resource as the female ages [see Additional file 2: Table S2]. The total intake of DM is converted into ME available for allocation, *ME*_*Acq*_, depending on the energy density of the DM available in the environment, *GE*_*Res*_ in Mcal/kg, and the metabolizability of the diet, *ME*_*PctGE*_, representing the energy losses through faeces, urine and enteric methane during digestion. It is affected by the level of dry matter intake and the proportion of concentrate as proposed by [16].

##### Utilization and physiological status sub-models

The detailed description of utilization and physiological status sub-models is in Additional file 4. The energy utilization sub-model combines ME from the acquisition sub-model with partition coefficients from the allocation sub-model and simulates the conversion of the energy, which is allocated to physiological functions (growth, gestation, lactation and somatic functions), into traits. The quantity of energy allocated to growth is converted into structural mass, which corresponds to the non-labile part of the body mass. The quantity of energy allocated to somatic functions is primarily used for maintenance and the remainder used for body reserves. The quantity of energy allocated to body reserves is converted into labile mass. This body compartment can subsequently be used to provide energy through mobilization, contrary to the structural mass. The quantity of energy allocated to gestation is converted into gravid uterus mass. Finally, the quantity of energy allocated to lactation is converted into milk production. Traits resulting from this conversion of energy into kg of matter are further used by the utilization sub-model to compute survival probability and conception probability, which are used in the physiological status sub-model. We assumed that survival probability became null when the female was not able to cover its maintenance requirements during 15 consecutive days or when it was selected for culling. Culling occurred after the second lactation, if conception did not occur 200 days after calving. Based on [17], we assumed that probability of conception is influenced by milk production, body condition score and energy balance.

#### Model calibration

The aim of this study was to evaluate the behaviour of the model in response to the variation of two parameters related to acquisition (*AcqB*_*GEN*_ and *AcqL*_*GEN*_) and of two parameters related to allocation (*G*2*S*_*GEN*_ and *Pc*2*S*_*GEN*_). Consequently, all other parameters were set at fixed values during the simulations. The values of parameters related to animal nutrition (diet characteristics and conversion of energy into body mass components and milk production) and reproduction (timing of events and probability of conception) were taken from previously published data based on the analysis of large datasets [see Additional file 1: Table S1]. The values of parameters related to dynamic and structural aspects of the model were determined during a calibration step. For some aspects such as the rates of transfer of priority, the model’s parameters cannot be measured directly from experimental data, but they can be inferred from data that track all the relevant traits throughout the animal’s lifespan. Unfortunately, very few studies have reported time series values for the full set of body mass, body reserves, milk production, and gestation mass through both young and adult phases of life. To overcome this limitation, it is possible to piece together consistent lifetime curves from a large number of suitably chosen studies over shorter time periods using a meta-analysis approach. This was done in a previously reported study to calibrate another model of dairy cow performance, called GARUNS [18, 19]. This GARUNS model is not suited to our current study on the dissociation of allocation and acquisition, but in the latter study, its use provided reference trajectories throughout life for the above-mentioned traits that represent the average curves from the literature. Accordingly, we used the GARUNS reference trajectories to calibrate the current model. Detailed aspects of the calibration are in Additional file 5. The comparison of the body mass, milk production and body condition score trajectories that were simulated by the model in the current paper, the GARUNS reference curves and the compilation of data from the literature are presented in Figures S10, S11 and S12 [see Additional file 6: Figures S10, S11 and S12]. The calibration step was done by iterative changes in parameter values until the model’s simulated trajectories converged with the GARUNS reference curves, and the data from the literature.

Finally, we evaluated the impact of the stochastic processes associated with the simulation of reproduction events. The use of a probability of conception *P*_*CONC*_ to determine if the simulated female becomes pregnant implies the use of a random process (see *CONCEPTION* event in Table S3 [see Additional file 3: Table S3]). As a result, for the same model parameterization, the time at which conception occurs, resulting from the random process, can vary among simulations and lead to slightly different outputs. To account for this stochastic aspect and stabilize the variance of the model’s outputs, each simulation had to be replicated 20 times.

#### Model simulations for sensitivity analysis

Model behaviour was explored by a global sensitivity analysis that aimed at evaluating how variation in model inputs, i.e. the four genetic-scaling parameters, affects FE. Two FE definitions were used, one at the lactation level and one at the end of the animal’s life. FE_Lac2 corresponds to the ratio between energy acquired and energy produced in milk, cumulated over the second lactation. FE_life corresponds to the same ratio, cumulated from birth to death. The four parameters (*G*2*S*_*GEN*_, *Pc*2*S*_*GEN*_, *AcqB*_*GEN*_ and *AcqL*_*GEN*_) were set at three different levels (L: low; M: medium and H: high) and combined in a complete factorial design [see Additional file 6: Table S4]. This led to 81 simulations, with 20 replications to account for the stochastic processes of reproduction. The discretization of parameters into levels allowed a reasonable computation time while enabling the exploration of the model behaviour in response to different combinations of values for genetic-scaling parameters. For each of the four genetic-scaling parameters, the medium level corresponded to the value that was determined in the calibration step [see Additional file 1: Table S1]. Values for low and high levels of parameters corresponded to equidistant deviations in percentage of the medium level. The percentages of deviations were chosen to simulate trajectories of traits that were consistent with the range of trait values observed in the existing data. The detailed description of the parameters levels is in Additional file 6. By testing all the combinations of parameter values, different individual profiles of acquisition and allocation were simulated and the corresponding lifetime trajectories of traits were used to compute FE. The sensitivity of the model’s output to variation in inputs was evaluated with sensitivity indices based on variance decomposition: output variability is decomposed into the main effects of parameters and interactions. Given our factorial simulation design, analysis of variance is a natural method for this variance decomposition [20].

## Results

**Fig. 5:**
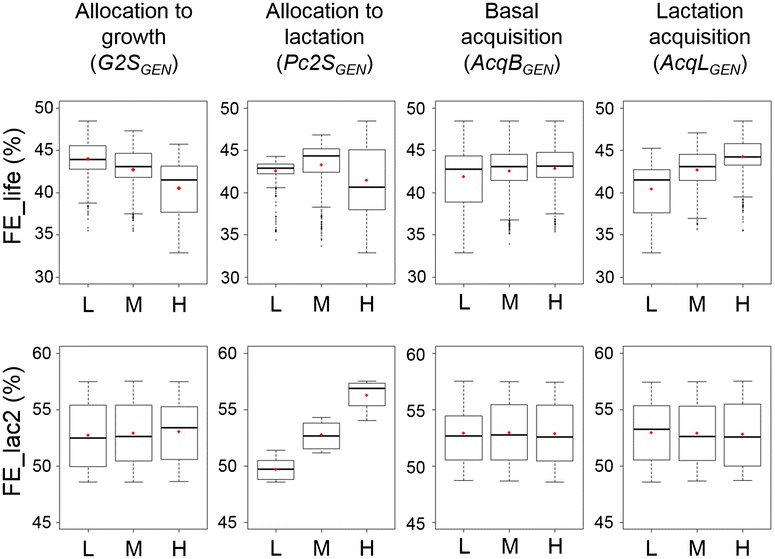
Results of the model sensitivity analysis for feed efficiency simulated at the lifetime level and second lactation level. FE_life, ratio between cumulative energy for milk production and cumulative energy acquired, from birth to death; FE_lac2, ratio between cumulative energy for milk production and cumulative energy acquired, from second parturition to second drying-off. The sensitivity analysis was based on a complete factorial design combining three levels (L: low; M: medium and H: high) of the four genetic-scaling parameters that drive allocation to growth (*G*2*S*_*GEN*_), allocation to lactation (*Pc*2*S*_*GEN*_), basal acquisition (*AcqB*_*GEN*_) and lactation acquisition (*AcqL*_*GEN*_). *Red dots* represent the mean values

The model simulates credible lifetime trajectories of acquisition and allocation and is sensitive to changes in genetic-scaling parameters, as shown in Additional file 6. Figure 5 shows the boxplots for the two definitions of FE. Outputs related to body mass, energy utilization and reproductive performance were also computed for each simulated cow, at the lactation level and at the end of life. Table 1 summarises the results for the two FE criteria and for the energy acquired and allocated to milk. Table 2 summarises outputs at the lactation level and Table 3 at the lifetime level. The analysis of variance used to compute sensitivity indices for the two definitions of FE is in Additional file 6.

**Table 1:** Measures of efficiency, energy acquired, and milk energy output for model sensitivity analysis

	Growth allocation			Lactation allocation			Basal acquisition			Lactation acquisition		
	L	M	H	L	M	H	L	M	H	L	M	H
FE_lac2												
Value (%)	52.70	52.92	53.04	49.65	52.76	56.24	52.87	52.94	52.84	52.95	52.88	52.82
Deviation from M (%)	−0.41	0.00	0.23	−5.90	0.00	6.61	−0.13	0.00	−0.20	0.13	0.00	−0.12
E_acq_lac2												
Value (10^3^ MJ)	54.58	54.66	54.84	54.70	54.67	54.71	51.55	54.68	57.85	52.49	54.68	56.90
Deviation from M (%)	−0.15	0.00	0.33	0.05	0.00	0.08	−5.72	0.00	5.81	−4.00	0.00	4.05
E_milk_lac2												
Value (10^3^ MJ)	28.75	28.92	29.09	27.15	28.84	30.77	27.25	28.95	30.56	27.80	28.92	30.05
Deviation from M (%)	−0.58	0.00	0.59	−5.84	0.00	6.71	−5.85	0.00	5.59	−3.87	0.00	3.93
FE_life												
Value (%)	43.97	42.71	40.55	42.53	43.27	41.43	41.86	42.51	42.85	40.38	42.62	44.23
Deviation from M (%)	2.96	0.00	−5.04	−1.72	0.00	−4.27	−1.54	0.00	0.80	−5.26	0.00	3.79
E_acq_life												
Value (10^3^ MJ)	636.20	559.59	464.99	708.03	573.46	379.28	481.09	557.99	621.69	452.51	551.67	656.60
Deviation from M (%)	13.69	0.00	−16.91	23.47	0.00	−33.86	−13.78	0.00	11.42	−17.98	0.00	19.02
E_milk_life												
Value (10^3^ MJ)	281.99	242.72	194.09	302.90	252.88	163.03	207.05	241.76	269.98	187.40	238.70	292.70
Deviation from M (%)	16.18	0.00	−20.03	19.78	0.00	−35.53	−14.36	0.00	11.67	−21.49	0.00	22.63

Measures are for the periods from birth to death (_life), and from parturition to drying off in second lactation (_lac2) for the three levels (L: low; M: medium and H: high) of the four genetic parameters that drive allocation to growth (*G*2*S*_*GEN*_), allocation to lactation (*Pc*2*S*_*GEN*_), basal acquisition (*AcqB*_*GEN*_) and lactation acquisition (*AcqL*_*GEN*_)

Values are in absolute terms and as percentages of the M level of the corresponding parameter

Parameters values are in Additional file 1: Table S1

**Table 2:** Outputs of model sensitivity analysis at the second lactation level

	Growth allocation			Lactation allocation			Basal acquisition			Lactation acquisition		
	L	M	H	L	M	H	L	M	H	L	M	H
Age at second parturition (days)	1138	1163	1225	1144	1164	1218	1214	1167	1145	1202	1172	1153
Interval between parturitions 2–3 (days)	406	418	423	386	404	455	409	419	417	429	413	404
E_grow_lac2 (10^3^ MJ)	0.501	0.544	0.544	0.568	0.541	0.480	0.450	0.538	0.600	0.476	0.533	0.579
E_mobilized_lac2 (% total energy use during lac2)	1.54	1.74	2.13	1.61	1.65	2.14	1.83	1.80	1.78	2.21	1.74	1.47
Labile mass at drying 2 (kg)	137	124	115	157	125	94	113	124	139	110	125	141

Outputs correspond to measures of age at second parturition, interval between second and third parturitions, cumulative energy for growth, cumulative energy mobilized for the period between parturition and drying-off of the second lactation and labile mass at the second drying for the three levels (L: low; M: medium and H: high) of the four genetic-scaling parameters that drive allocation to growth (*G*2*S*_*GEN*_), allocation to lactation (*Pc*2*S*_*GEN*_), basal acquisition (*AcqB*_*GEN*_) and lactation acquisition (*AcqL*_*GEN*_)

**Table 3:** Outputs of model sensitivity analysis at the lifespan level

	Growth allocation			Lactation allocation			Basal acquisition			Lactation acquisition		
	L	M	H	L	M	H	L	M	H	L	M	H
Longevity (years)	11.3	10.2	8.8	12.4	10.4	7.6	9.6	10.2	10.6	8.9	10.1	11.3
Productive longevity (years)	7.7	6.7	5.4	8.6	6.8	4.3	6.1	6.7	7.0	5.5	6.6	7.7
Number of lactations	8.8	7.6	6.1	9.9	7.8	4.9	6.9	7.6	8.0	6.3	7.5	8.7
Structural mass (kg)	425	446	468	446	446	446	406	446	487	446	446	446
Mean labile mass at drying (kg)	138	126	114	160	126	93	113	126	140	109	126	144
E_grow_life (10^3^ MJ)	10.24	10.79	11.37	10.83	10.81	10.76	9.80	10.81	11.80	10.69	10.80	10.92
E_balance_life (10^3^ MJ)	12.81	11.99	10.88	15.49	11.71	8.47	10.82	11.89	12.97	10.51	11.78	13.38
E_maintenance (% total energy expenditure)	48.30	49.09	50.44	49.58	48.73	49.51	49.70	49.22	48.91	50.64	49.14	48.05
E_mobilized_life (% total energy use)	1.59	1.63	1.70	1.82	1.52	1.57	1.64	1.63	1.63	1.74	1.59	1.58
E_milk_lactation (10^3^ MJ)	31.92	31.69	31.15	30.54	31.87	32.36	29.63	31.62	33.51	29.73	31.60	33.43

Outputs correspond to measures of longevity, productive longevity, number of lactations, structural mass, mean labile mass at drying-off and cumulative energy outputs for the period between birth and death for the three levels (L: low; M: medium and H: high) of the four genetic-scaling parameters that drive allocation to growth (*G*2*S*_*GEN*_), allocation to lactation (*Pc*2*S*_*GEN*_), basal acquisition (*AcqB*_*GEN*_) and lactation acquisition (*AcqL*_*GEN*_). Energy outputs are: cumulative energy for growth, total energy balance (difference between cumulative energy for reconstitution of reserves and cumulative energy mobilized), cumulative energy mobilized and average cumulative energy per lactation. Parameters values are in Additional file 1: Table S1

### Allocation to growth: sensitivity of phenotypic traits to the variation in the genetic-scaling parameter *G*2*S*_*GEN*_

#### Lactation level

During second lactation, increasing allocation to growth had a negligible positive effect on FE_lac2 (Fig. 5). It also had a negligible positive effect on cumulative energy allocated to milk production (E_milk_lac2 in 10^3^ MJ) and on cumulative energy acquired during second lactation (E_acq_lac2 in 10^3^ MJ) as shown in Table 1. Changes in E_milk_lac2 for different levels of allocation to growth (−0.58 and +0.59 % for L and H compared to M) were larger than changes in energy acquired (−0.15 and +0.33 % for L and H compared to M). As a result, the ratio FE_lac2 slightly increased. The small increase in E_acq_lac2 for increasing allocation to growth was due to an increase in age at second parturition (Table 2). When individuals were older at parturition, they also had a higher level of basal acquisition because it increased with age, until maturity. The slight increase in E_milk_lac2 for increasing allocation to growth was due to an increase in the interval between second and third parturitions (Table 2). Increasing allocation to growth resulted in a decrease in labile mass at the second drying (from 137 to 115 kg, see Table 2), and consequently a delay in the time to next gestation. When gestation occurred later, the period during which this function coexisted with lactation decreased. In the model, allocation to gestation decreased allocation to lactation (see Equation E2 in Table S2 [see Additional file 2: Table S2]). The shorter the period of coexistence was, the lower was the depressive effect of gestation on lactation, allowing a slight increase in E_milk_lac2. Regarding the energy used for growing during second lactation (E_grow_lac2 in 10^3^ MJ), increasing allocation to growth led to very small differences in energy expenditure for growth (Table 2). As most of the energy for growing was spent before second lactation, the different levels of allocation to growth led to only small differences during second lactation.

#### Lifetime level

Increasing allocation to growth resulted in a decrease in FE_life (Fig. 5) associated with a decrease in energy used for milk production (E_milk_life in 10^3^ MJ) and a decrease in energy acquired (E_acq_life in 10^3^ MJ). As shown in Table 1, changes in E_milk_life were greater than changes in E_acq_life, leading to a decreased ratio FE_life. The two components of FE_life both decreased because the increase in allocation to growth led to a shorter lifespan and a shorter productive life with a smaller number of lactations (Table 3). Increasing growth allocation had negative effects on survival and reproduction, which were due to the trade-off between structural mass and labile mass. In the model, the energy allocated to growth fuels the structural mass rather than the labile mass, which is fuelled by energy allocated to somatic functions. Consistently, an increase in allocation to growth led to an increase in structural mass (Table 3) because the energy used for growth increased (E_grow_life in 10^3^ MJ). At the same time, an increase in allocation to growth led to a decrease in labile mass, as illustrated in Table 3 by the mean labile mass at drying. Furthermore, it led to an increase in labile mass mobilization as shown by the proportion of energy mobilized (Table 3). The level and the use of labile mass are involved in survival (ability to cover maintenance requirements) and reproduction (effect of body condition score and energy balance on conception probability). As a result, the ratio between productive lifespan and longevity decreased from 0.68 to 0.61 as allocation to growth increased, which increased the proportion of energy spent for maintenance over the lifetime (E_maintenance in % of total energy expenditure, Table 3), thus reducing the dilution of maintenance costs and decreasing FE.

### Allocation to lactation: sensitivity of phenotypic traits to variation in the genetic-scaling parameter *Pc*2*S*_*GEN*_

#### Lactation level

During second lactation, increasing allocation to lactation strongly increased FE_lac2 (Fig. 5). This effect was due to an increase in E_milk_lac2 and a stagnation of E_acq_lac2 (Table 1). The increase in energy used for milk was made possible by a higher mobilization of body reserves (Table 2).

#### Lifetime level

Contrary to effects at the lactation level, increasing allocation to lactation was not beneficial at the lifetime level (Fig. 5). When *Pc*2*S*_*GEN*_ increased from L to M, FE_life increased slightly. When it increased from M to H, FE_life decreased. When allocation to lactation increased, both E_acq_life and E_milk_life decreased. On the one hand, the increase in allocation to lactation from L to M led to a larger decrease in E_acq_life than in E_milk_life, and thus to a slight increase in FE_life. On the other hand, the increase in allocation to lactation from M to H led to a smaller decrease in E_acq_life than in E_milk_life, thus to a decrease in FE_life. Increasing allocation to lactation decreased E_acq_life and E_milk_life (Table 1). These effects were modulated by a decrease in the number of lactations associated to shorter lifespan and productive life (Table 3). This effect was due to a lower labile mass at drying, which impaired survival and reproductive performance. This lower labile mass was not linked to growth, i.e. the structural mass was similar for all levels of allocation to lactation. Within the full factorial design, variations of growth allocation and basal acquisition, which both determine structural mass, were the same across various levels of allocation to lactation. This lower labile mass was due to a decrease in life energy balance (E_balance_life, defined as the cumulative energy for labile repletion minus the cumulative energy for mobilization in 10^3^ MJ) when allocation to lactation increased. In spite of the overall decrease in E_acq_life and E_milk_life that is caused by a shorter life, increasing allocation to lactation logically led to an increase in the energy used for milk production per lactation (E_milk_lactation in 10^3^ MJ). When increasing allocation to lactation from L to M, the higher productivity per lactation compensated the decrease in lifespan and productive life. The proportion of energy spent on maintenance decreased slightly from L to M (Table 3), which improved the dilution of maintenance costs and increased FE_life. When increasing allocation to lactation from M to H, the higher productivity per lactation did not compensate the reduction in lifespan and productive life (the ratio productive life/longevity dropped from 0.65 to 0.57). The proportion of energy spent on maintenance increased, which resulted in less dilution of maintenance costs and a decrease in FE_life.

### Basal acquisition: sensitivity of phenotypic traits to variation in the genetic-scaling parameter *AcqB*_*GEN*_

#### Lactation level

During second lactation, increasing basal acquisition had almost no effect on FE_lac2 (Fig. 5). Increasing basal acquisition increased E_acq_lac2 and E_milk_lac2 in the same proportion (Table 2), thus leading to the same ratio.

#### Lifetime level

Increasing basal acquisition led to a very small increase in FE_life (Fig. 5). Both E_acq_life and E_milk_life increased substantially when basal acquisition increased. This effect was due to a small increase in labile mass made possible by the increased acquisition (Table 3), which favoured reproduction and therefore productive life. As a result, energy for maintenance represented a slightly smaller part of the energy budget (Table 3). This slight improvement in dilution of maintenance costs explained the small increase in FE_life.

### Lactation acquisition: sensitivity of phenotypic traits to variation in the genetic-scaling parameter *AcqL*_*GEN*_

#### Lactation level

During second lactation, increasing lactation acquisition had almost no effect on FE_lac2 (Fig. 5). As for basal acquisition, increasing lactation acquisition increased E_acq_lac2 and E_milk_lac2 in the same proportion (Table 1), thus leading to the same ratio.

#### Lifetime level

Contrary to the lactation level, increasing lactation acquisition resulted in a substantial increase in FE_life (Table 1). Both E_milk_life and E_acq_life increased when lactation acquisition increased but E_milk_life increased proportionally more than E_acq_life, thus increasing the ratio. The overall increase in E_milk_life and E_acq_life was due to an increase in lifespan and productive life, because of a larger number of lactations. Increasing lactation acquisition led to a higher level of labile mass, which favoured survival and reproduction (the ratio between productive life and longevity increased from 0.62 to 0.68). With a longer lifespan and more lactations, the energy spent on maintenance represented a smaller part of the energy budget (50.64, 49.14 and 48.05 % for L, M and H), which improved the dilution of fixed costs and increased FE_life.

## Discussion

### Effects of acquisition and allocation on feed efficiency

Our simulation results show that the mechanisms of acquisition and allocation had contrasted effects on FE depending on the time scale over which efficiency was calculated, as shown in Fig. 5. At the lactation level, improvement in FE was achieved by increasing allocation to lactation. This result is consistent with past selection strategies on milk production, leading to a dilution of maintenance costs [21]. At the lifetime level, improvement in FE was achieved by decreasing allocation to growth and increasing lactation acquisition. This improvement was caused by a higher level of body reserves (see Sections Allocation to growth: sensitivity of phenotypic traits to the variation in the genetic-scaling parameter *G2S*_*GEN*_ and Lactation acquisition: sensitivity of phenotypic traits to variation in the genetic-scaling parameter *AcqL*_*GEN*_), which resulted in improved reproduction and survival and thus favoured the dilution of the non-productive part of the lifespan. In contrast to the effect at the lactation level, increasing allocation to lactation improved lifetime FE up to an optimal level, beyond which the effect on FE became negative.

These findings highlight the crucial role of life stages when considering selection for FE and the metric associated with the phenotype selected for. Based on our simulation results, selecting for FE at the lactation level will result in the selection of females with high allocation to lactation. These females are not those that maximize FE at the lifetime level, i.e. short-term efficiency may come at the cost of reduced sustainability. In contrast, selecting for FE at the lifetime level will result in the selection of females with low allocation to growth and high lactation acquisition. These females are not those that maximize FE at the lactation level. The importance of the life stages in interpreting efficiency measures was previously mentioned by [3].

The lifetime effects of acquisition and allocation mechanisms on FE were mainly due to the central role of body reserves. This component is pivotal in the phenotypic feedback of acquisition and allocation on survival and reproduction. The impact of allocation to growth was due to a direct trade-off between structural mass and body reserves. Increasing allocation to growth decreased body reserves, which had a negative effect on survival and reproduction. The impact of lactation acquisition on FE was due to a positive effect on body reserves. Increasing acquisition, with equivalent maintenance costs, resulted in larger body reserves, and thus had a positive effect on survival and reproduction. This emphasizes the importance of body reserves for animal resilience [22–24]. The feedback of body reserves on survival and reproduction led to differences in lifespan and number of production cycles. Such variations modulate the dilution of the fixed costs, through maintenance expenditure and non-productive stages, and therefore affect FE. The role of body reserves in improving FE is not straightforward, as shown by the lifetime effect of allocation to lactation on FE. Our results suggested an optimal level of allocation to lactation. Increasing allocation to lactation from a low to a medium level had a positive effect on FE in spite of reducing lifespan and lactation number. This result agrees with the recent work of [17] who reported that dairy cows with high genetic merit for milk have a shorter lifespan and lower reproductive performance, but they have a slightly higher lifetime FE than cows with low genetic merit. The higher level of production per lactation cycle compensated for the smaller number of lactation cycles. When allocation to lactation increased from a medium to a high level, this positive effect disappeared and FE decreased. The previous offset of production per lactation cycle was not sufficient to balance the reduction in number of lactation cycles. These results highlight the complexity of FE as a phenotype, which results from a combination of production time and production level. It is clear that improving FE implies the dilution of fixed costs linked to maintenance expenditure and non-productive stages. In the past, the strategy was based on paying back fixed costs with a higher level of production. However, this resulted in negative effects on other time components of FE (lifespan, reproductive success) and this strategy is not adapted to variable or low quality environments [25]. Thus, the challenge is to find the optimal strategy for the dilution of costs depending on the environmental conditions. In addition, more research is needed to quantify the genetic variability in the relation between body reserves and their effect on reproductive performance. This relation can greatly affect FE over the lifetime and therefore should be included in future selection strategies for FE. The model structure is flexible enough to incorporate such future findings. The parameters that determine how body condition score, milk and energy balance influence the probability of conception can be considered as genetic-scaling parameters and set at different values. Thus, as discussed in the following section, simulations will allow the comparison of FE associated with genotypes that reflect various effects of body reserves on reproductive performance.

### Modelling approach

We propose a model that demonstrated the relevance of breaking-down FE into acquisition and allocation mechanisms. They play different roles on FE depending on the female’s life-stage and this may impact future selection strategies. This alone justifies the conceptual break with the majority of the models for performance prediction that use production potential, i.e. the product of acquisition and allocation (to that output), as the genetic driver. In our model, the genetic-scaling parameters are dissociated into independent parameters that control acquisition and allocation separately. Thus, our model offers opportunities to explore different biological strategies of paying back fixed costs to improve FE, and to investigate which strategy is better adapted to a given environment. The idea here is to identify a point of diminishing returns, that is to say the point after which increasing lifespan or productivity no longer increases FE. Other genetic-scaling parameters could be explored (for instance shape of lactation curve or gestation allocation for prolific species) to further evaluate which combinations of acquisition and allocation maximize FE. Furthermore, other environments could be explored to better use the ability of the model to represent G × E interactions. In this study, we considered a constant environment with fixed resources, both in quantity and quality. The next step could be to carry out a sensitivity analysis of the model to different levels of energy density and evaluate if acquisition and allocation mechanisms have the same impact on feed efficiency. Using the model for various environments will raise the larger issue of available data for validation of the model components that simulate environmental effects. In this study, we validated the consistency of phenotypic trajectories (body mass, body condition score and milk production) simulated by the model with the trajectories corresponding to a compilation of data from the literature, and carried out a global sensitivity analysis, which are two key steps of the model validation [26, 27]. To go further than this partial validation, we will need data corresponding to frequent and long-term measurements and data related to feed intake, body mass and milk production. Even if not yet available, such datasets are likely to be provided by on-going projects on feed efficiency.

The model presented here represents a single cow, defined by its genetic-scaling parameters. By simulating different values of these parameters, the model allows the comparison of phenotypic performances expressed by different genotypes, which reflect different strategies of acquisition and allocation. Clearly, further exploration of the genetic aspects would require a population model. Using the techniques of individual-based modelling, the present animal model could be multiplied to create a virtual population within which each animal has its own genetic-scaling parameters. Such a population model could either specify additive genetic (co)variance structures among the genetic-scaling parameters, or let them (co)evolve naturally. In the present animal model, we broke the phenotypic correlations by decomposing mechanisms and then studied the effects of parameters that drive these mechanisms by setting independent values. In the population context, we need to re-introduce genetic correlations at the level of mechanisms (for instance, the correlation between the value of the growth allocation parameter and the value of the basal acquisition parameter). Using the model as the building block of an individual-based population model will also allow the incorporation of trans-generational aspects. By simulating populations of genotypes under selection, we will be able to evaluate which genetic-scaling parameters combinations are selected depending on environmental conditions and thus to improve the prediction of the effects of selection strategies in different environments.

## Conclusions

Feed efficiency is a complex phenotype, which in physiological terms combines the acquisition of resources from the environment with the allocation of resources between physiological functions, including production and non-productive functions. Our results show that breaking-down feed conversion into acquisition and allocation, and introducing genetically-driven trajectories that control these mechanisms, permitted quantification of their relative roles on feed efficiency. Furthermore, our results show that the life stage at which feed efficiency is evaluated appears to be a key aspect for selection. When feed efficiency is evaluated over the second lactation, it is mainly affected by allocation to lactation. When feed efficiency is evaluated in the long-term, i.e. over the whole lifespan, it is mainly affected by allocation to growth and acquisition of resource during lactation. While there is a strong linear increase in feed efficiency with more allocation to lactation within a lactation cycle, our results suggest that there is an optimal level of allocation to lactation beyond which increasing allocation to lactation negatively affects lifetime feed efficiency. Our modelling approach highlights the role of body reserves in the prediction of lifetime feed efficiency since they integrate the feedback of acquisition and allocation on survival and reproduction. It also provides new insights into the processes that underpin lifetime feed efficiency in dairy cows.

## Additional files

**Additional file 1: Table S1.** Parameters of the different sub-models. The table provides the listing of the symbols used for the model’s parameters, the description of the parameters, their values and the source used to define these values [16–18, 28].

**Additional file 2: Table S2.** Elements and equations of the allocation, acquisition and utilization sub-models. The table provides the listing of the elements included in the model (compartments, flows, variables) with their symbols and units. It also provides the equations that define them or indicate the discrete events that change their values [16–19, 29].

**Additional file 3: Table S3.** Description of the discrete events in the physiological sub-model. Description: The table provides the description of the triggers that activate the discrete events and the actions on model’s elements implemented by the events.

**Additional file 4.** Details of model description. Description: The document provides the description of all model’s equations and detailed aspects of the discrete events functioning. Figure S1 shows an example of the flows of energy simulated by the model [15–17, 28–30].

**Additional file 5.** Details of the calibration of the model. The document provides a description of some detailed aspects of the model’s calibration. It indicates the assumptions that were made to introduce biological relationships in the model and how the values of the parameters for these relationships were defined. The relationships relate to the effect of the degree of maturity on the energetic value of structural mass gain and growth function efficiency, the proportion of energy allocated to gestation (Figure S2), the age-dependence of the energetic value for maintenance (Figures S3, S4 and S5), the effect of parity on lactation acquisition (Figures S6, S7 and S8) and the effect of parity on lactation allocation (Figure S9) [18, 19, 31, 32].

**Additional file 6.** Calibration results and sensitivity analysis details. The document provides the results of the calibration step in the model development. This step was based on a comparison with the GARUNS model and completed by a comparison with a compilation of data from literature related to body mass (Figure S10), milk production (Figure S11) and body condition score (Figure S12). The document also provides additional information on the global sensitivity analysis with the values of the parameters used in the complete factorial design (Table S4) and on how these values of the parameters were determined. Finally, the document provides additional results of the sensitivity analysis with the variance decomposition of the two feed efficiencies depending on the input parameters (Table S5) and series of figures that show the effects of parameters on the dynamic changes of empty body mass, dry matter intake and milk production (Figures S13, S14, S15, S16, S17, S18, S19, S20, S21, S22, S23 and S24) [18, 19, 33–41].
