# Supplementary material for: Disentangling the relative roles of resource acquisition and allocation on animal feed efficiency: insights from a dairy cow model
Source: Genet Sel Evol. 2016 Sep 26;48:72. doi: 10.1186/s12711-016-0251-8 (PMC5037647; doi:10.1186/s12711-016-0251-8)
Supplement: Supplementary file 1 — Additional file 1: Table S1. Parameters of the different sub-models. The table provides the listing of the symbols used for the model’s parameters, the description of the parameters, their values and the source used to define these values [16–18, 28]. [file 12711_2016_251_MOESM1_ESM.docx]

**Additional file 1 Table S1 Parameters of the different sub-models**

| **Symbol** | **Description** | **Value** | **Source** |
| --- | --- | --- | --- |
| Allocation sub-model |  |  |  |
| $G2S_{GEN}$ | Genetic-scaling parameter for priority transfer from growth to somatic functions | 0.00265^*^ | Calibration |
| $Pc2S_{GEN}$ | Genetic-scaling parameter for priority transfer from lactation to somatic functions | 0.0016^*^ | Calibration |
| $k_{H}Pf_{0}$ | Parameter for priority transfer from somatic functions to future progeny (see Section “Calibration of the proportion of energy allocated to gestation, representing allocation for future progeny $AllocPf$“ in Additional file 5) | 0.000001 | Calibration |
| $k_{H}Pf_{1}$ |  | 0.18 |  |
| $k_{H}Pf_{2}$ |  | 6 |  |
| $k_{H}Pf_{3}$ |  | 180 |  |
| $k_{H}Pf_{4}$ |  | $PARTURITION$ event |  |
| $k_{G0}$ | Initial value of allocation to growth | 0.46 | Calibration |
| $k_{init}Pc_{1}$ | Effect of parity on the charge of AllocS_2_ at parturition (see Section “Effects on parity on lactation allocation” in Additional file 5) | 0.34 | Calibration |
| $k_{init}Pc_{2}$ |  | 0.8 |  |
| $k_{init}Pc_{3}$ |  | 0.032 |  |
| $k_{init}Pc_{4}$ |  | 0.27 |  |
| $S2Pc$ | Parameter for priority transfer from somatic functions to current progeny | 0.2 | Calibration |
| Acquisition sub-model |  |  |  |
| $AcqB_{GEN}$ | Genetic-scaling parameter for basal acquisition (kg DM/d) | 11.5^*^ | Calibration |
| $AcqL_{GEN}$ | Genetic-scaling parameter for lactation acquisition (kg DM/d) | 8.5^*^ | Calibration |
| $k_{AcqB_{Mat}}$ | Equation of basal acquisition maturation (kg DM/d) | 0.0025 | Calibration |
| $k_{M}AcqL_{1}$ | Effect of parity on the maximum of the acquisition curve during lactation (see Section “Effects of parity on lactation acquisition” in Additional file 5) | 1.2 | Calibration |
| $k_{M}{AcqL}_{2}$ |  | 0.24 |  |
| $k_{M}{AcqL}_{3}$ |  | 0.076 |  |
| $k_{M}AcqL_{4}$ |  | 0.3 |  |
| $k_{D}AcqL_{1}$ | Shape of lactation acquisition curve (see Section “Effects of parity on lactation acquisition” in Additional file 5) | $PARTURITION$ event | Calibration |
| $k_{D}AcqL_{2}$ |  | 0.06 |  |
| $k_{D}AcqL_{3}$ |  | $PARTURITION$ event |  |
| $k_{D}AcqL_{4}$ |  | 0.72 |  |
| $GE_{Res_{Ref}}$ | Reference value for diet gross energy density (MJ/kg DM) | 21.275 | [16] |
| $ME_{pctGE_{Ref}}$ | Reference value for diet metabolizability (MJ NE/MJ ME) | 0.578 |  |
| $C{O_{Res}}_{Ref}$ | Reference value for diet proportion of concentrate feedstuff (kg concentrate/kg DM) | 0.4 |  |
| $ND{F_{Res}}_{Ref}$ | Reference value for diet proportion of fibers (kg fibers/kg DM) | 0.384 | [29] |
| Physiological sub-model |  |  |  |
| $Len_{Gest}$ | Gestation length (days) | 280 | [17] |
| $Len_{WaitPer}$ | Delay between parturition and conception (days) | 60 |  |
| $Len_{Lac}$ | Lactation length (days) | 320 |  |
| $Len_{Oest}$ | Oestrus length (days) | 22 |  |
| Energy utilization sub-model |  |  |  |
| $EV_{GainUterus}$ | Energy value (MJ/kg) of 1 kg of gravid uterus | 29.288 | [18] |
| $EV_{GainLabile}$ | Energy value (MJ/kg) of 1 kg of labile mass gain | 31.380 |  |
| $EV_{LossLabile}$ | Energy value (MJ/kg) of 1 kg of labile mass loss | 31.380 |  |
| $EV_{Milk}$ | Energy value (MJ/kg) of 1 kg of milk | 3.134 |  |
| ${EFF}_{Mnt}$ | Efficiency of maintenance (MJ of NE/MJ of ME) | 0.65 |  |
| ${EFF}_{GainLabile}$ | Efficiency of labile mass gain (MJ of NE/MJ of ME) | 0.67 |  |
| ${EFF}_{LossLabile}$ | Efficiency of labile mass loss (MJ of NE/MJ of ME) | 0.82 |  |
| ${EFF}_{Lac}$ | Efficiency of lactation (MJ of NE/MJ of ME) | 0.64 |  |
| $H_{EVMnt0}$ | Effect of age on the energetic cost of maintaining 1 kg of metabolic weight (see Section “Age dependence of the energetic value for maintenance EV_Mnt” in Additional file 5) | 0.389 | Calibration |
| $H_{EVMnt1}$ |  | 2.092 |  |
| $H_{EVMnt2}$ |  | 3 |  |
| $H_{EVMnt3}$ |  | 11250 |  |
| $Mass_{Birth}$ | Body mass at birth in kg | 45 | [18] |
| $k_{Labile_{Birth}}$ | Proportion of labile mass at birth | 0.07 |  |
| $Ratio_{GainStruct}$ | Parameter of the equation for theoretical structural mass, $Mas{s_{Struct}}_{theo}$ (see Section “Calibration of the proportion of energy allocated to gestation, representing allocation for future progeny $AllocPf$“ in Additional file 5) | 0.03754409 | Calibration |
| $Mas{s_{Struct}}_{theo}$ | Theoretical structural mass, obtained for growth in a reference nutritional environment (see Section “Calibration of the proportion of energy allocated to gestation, representing allocation for future progeny $AllocPf$“ in Additional file 5) | 442.7 |  |
| $C_{MassStruct_{theo}}$ | Parameter of the equation for theoretical structural mass, $Mas{s_{Struct}}_{theo}$ ((see Section “Calibration of the proportion of energy allocated to gestation, representing allocation for future progeny $AllocPf$“ in Additional file 5) | 0.2124 |  |

^*^: values from the calibration step used as the medium level in the complete factorial design simulated for the sensitivity analysis.
