# Supplementary material for: Disentangling the relative roles of resource acquisition and allocation on animal feed efficiency: insights from a dairy cow model
Source: Genet Sel Evol. 2016 Sep 26;48:72. doi: 10.1186/s12711-016-0251-8 (PMC5037647; doi:10.1186/s12711-016-0251-8)
Supplement: Supplementary file 2 — Additional file 2: Table S2. Elements and equations of the allocation, acquisition and utilization sub-models. The table provides the listing of the elements included in the model (compartments, flows, variables) with their symbols and units. It also provides the equations that define them or indicate the discrete events that change their values [16–19, 29]. [file 12711_2016_251_MOESM2_ESM.docx]

**Additional file 2 Table S2 Elements and equations of the allocation, acquisition and utilization sub-models**

| **Symbol** | **Definition** | **Equation (or event relation)** | **Equation nb** |
| --- | --- | --- | --- |
| **Allocation sub-model** | | |  |
| $f_{prio}G2S$ | Priority transfer from growth to survival | $f_{prio}G2S=AllocG*(G2S_{GEN}+0.01*AllocPf)$ | E1 |
| $f_{prio}Pc2S$ | Priority transfer from current progeny to survival | $f_{prioPc2S}=AllocPc\cdot Lac\_Stat\cdot(Pc2S_{GEN}+AllocPf\cdot Gest_{Stat}\cdot0.06)$ | E2 |
| $f_{prio}S2Pf$ | Priority transfer from survival to future progeny | $f_{prio}S2Pf =Gest_{Stat}\cdot\left( k_{H}Pf_{1}+k_{H}Pf_{4}-k_{H}Pf_{0} \right)\cdot(e^{-k_{H}Pf_{2}\cdot\ln\left( k_{H}Pf_{3} \right)}\cdot k_{H}Pf_{2}\cdot{Gest_{Time}}^{(k_{H}Pf_{2}-1)}\cdot\frac{1}{\left( 1+e^{-k_{H}Pf_{2}\cdot ln(k_{H}Pf_{3})}\cdot{Gest_{Time}}^{k_{H}Pf_{2}} \right)^{2}}$ | E3^a^ |
| $f_{prio}S2Pc$ | Priority transfer from survival to current progeny | $f_{prio}S2Pc=AllocS_{2}\cdot S2Pc\cdot Lac\_Stat$ | E4 |
| $AllocG$ | Allocation to growth (proportion of ME acquired) | $\frac{dAllocG}{dt}=-f_{prio}G2S\cdot Alive_{Stat}$ | E5 |
| $AllocS$ | Allocation to somatic functions (proportion of ME acquired) | $\frac{dAllocS}{dt}=(f_{prio}G2S-f_{prio}S2Pf+f_{prio}Pc2S-f_{prio}S2Pc)\cdot Alive_{Stat}$ | E6 |
| $AllocPf$ | Allocation to future progeny (proportion of ME acquired) | $\frac{dAllocPf}{dt}=+f_{prio}S2Pf\cdot Alive_{Stat}$ | E7 |
| $AllocPc$ | Allocation to current progeny (proportion of ME acquired) | $\frac{dAllocPc}{dt}=(+f_{prio}S2Pc-f_{prio}Pc2S)\cdot Alive_{Stat}$ | E8 |
| $AllocS_{1}$ | Sub-compartment of allocation to somatic functions | $\frac{dAllocS_{1}}{dt}=(f_{prio}G2S -f_{prio}S2Pf+f_{prio}Pc2S)\cdot Alive_{Stat}$ | E9 |
| $AllocS_{2}$ | Sub-compartment of allocation to somatic functions | $\frac{dAllocS_{2}}{dt}=-f_{prio}S2Pc\cdot Alive_{Stat}$ | E10 |
| **Acquisition sub-model** | | |  |
| $AcqT$ | Total acquisition of DM (kg/day) | $AcqT=AcqB+AcqL$ | E11 |
| $AcqB$ | Basal acquisition of DM (kg/day) | $AcqB=(AcqB_{GEN}-0.8\cdot AcqB_{GEN} \cdot e^{-k_{AcqB_{MAT}}\cdot t})\cdot Alive_{Stat}$ | E12 |
| $AcqL$ | Lactation acquisition of DM (kg) | $AcqL=Lac_{Stat}\cdot Alive_{Stat}\cdot AcqL_{Max}\cdot AcqL_{Dyn}(Lac_{Time})$ | E13 |
| $AcqL_{Max}$ | Maximum DM intake reached during lactation (kg) | $AcqL_{Max}(Gest_{Nb})=AcqL_{GEN}\cdot AcqL_{pctMAT}(Gest_{Nb})$ | E14^b^ |
| $AcqL_{Dyn}$ | Dynamic change of acquisition during lactation (dimensionless) | $AcqL_{Dyn}\left( Lac_{Time} \right)=\left( \left( k_{D}AcqL_{1}\cdot\frac{k_{D}AcqL_{2}}{k_{D}AcqL_{3}-k_{D}AcqL_{2}}\cdot\left( e^{-Lac_{Time}\cdot k_{D}AcqL_{2}}-e^{-Lac_{Time}\cdot k_{D}AcqL_{3}} \right) \right)-k_{D}AcqL_{4} \right)$ | E15^b^ |
| $ME_{Acq}$ | ME acquired (MJ/day) | $ME_{Acq}= (AcqB+AcqL)\cdot GE_{Res}\cdot ME_{PctGE}$ | E16 |
| $ME_{PctGE}$ | Metabolizability of the nutritional resource (MJ NE/MJ ME) | $ME_{PctGE}= {(50.9-1.01\cdot\left( \frac{AcqB+AcqL}{\frac{Mass}{100}} \right)+16.35\cdot CO_{Res}+8.52\cdot CO_{Res}^{2})}/{100}$ | E17^c^ |
| $GE_{Res}$ | Gross energy density of the nutritional resource (MJ/kg) | $GE_{Res}=G{E_{Res}}_{Ref}$ | E18 |
| $NDF_{Res}$ | Proportion of fibers in the diet (kg NDF/kg DM) | $NDF_{Res}=ND{F_{Res}}_{Ref}$ | E19 |
| $CO_{Res}$ | Proportion of concentrate feedstuff in the diet (kg CO/kg DM) | $CO_{Res}=C{O_{Res}}_{Ref}$ | E20 |
| **Utilization sub-model** | | |  |
| $ME_{Growth}$ | ME for growth( MJ/day) | $ME_{Growth}(t)= AllocG\cdot ME_{Acq}$ | E21 |
| $ME_{GravidUterus}$ | ME for gestation (MJ/day) | $ME_{GravidUterus}=AllocPf\cdot ME_{Acq}$ | E22 |
| $ME_{Milk}$ | ME for milk production (MJ/day) | $ME_{Milk}=AllocPc\cdot ME_{Acq}$ | E23 |
| $ME_{Soma}$ | ME for somatic functions (MJ/day) | $ME_{Soma}=AllocS\cdot ME_{Acq}$ | E24 |
| $ME_{MntReq}$ | ME requirement for maintenance (MJ/day) | $ME_{MntReq}={EV_{Mnt}\cdot{Mass}^{0.75}}/{EFF_{Mnt}}$ | E25 |
| $ME_{VarStock}$ | Variation in energy balance (MJ/day) | $ME_{VarStock}=ME_{Soma}-ME_{MntReq}$ | E26 |
| $ME_{LabileRep}$ | ME for body reserves repletion (MJ/day) | $ME_{LabileRep}=VarStock_{Stat}\cdot ME_{VarStock}$ | E27 |
| $ME_{LabileMob}$ | ME from body reserves mobilization (MJ/day) | $ME_{LabileMob}=min\left( \left( \left( 1-VarStock_{Stat} \right)\cdot-ME_{VarStock} \right),(Mass_{Labile}\cdot EV_{LossLabile}\cdot Eff_{LossLabile}) \right)$ | E28 |
| $ME_{Mnt}$ | ME for maintenance (MJ/day) | $ME_{Mnt}=ME_{Soma}-ME_{LabileRep}+ME_{LabileMob}$ | E29 |
| $ME_{MntDeficit}$ | Deficit of maintenance requirement coverage (proportion) | $ME_{MntDeficit}=(ME_{Mnt}-ME_{MntReq})/ME_{MntReq}$ | E30 |
| $Acc_{MntDef}$ | Accumulation of deficit to cover maintenance requirements | $\frac{dAcc_{MntDef}}{dt}=ME_{MntDef}$ | E31 |
| $Labile_{A}$ | Labile mass repleted (kg/day) | $Labile_{A}={ME_{VarStock}\cdot VarStock_{Stat}\cdot EFF_{GainLabile}}/{EV_{GainLabile}}$ | E32 |
| $Labile_{C}$ | Labile mass mobilization (kg/day) | $Labile_{C}=min\left( \left( 1-VarStock_{Stat} \right)\cdot-ME_{VarStock}\cdot\frac{1}{EFF_{LossLabile}}\cdot\frac{1}{EV_{LossLabile}},Mass_{Labile} \right)$ | E33 |
| $Struct_{A}$ | Structural mass gain (kg/day) | $Struct_{A}=ME_{Growth}{\cdot EFF_{GainStruct}}/{EV_{GainStruct}}$ | E34 |
| $Mass_{Uterus}$ | Uterus mass (kg) | $\frac{dMass_{Uterus}}{dt}=AllocPf\cdot ME_{Acq}\cdot Gest_{Stat}\cdot\frac{1}{EV_{GainUterus}}$ | E35 |
| $Mass_{Labile}$ | Labile mass (kg) | $\frac{dMass\_Labile}{dt}=Labile_{A}-Labile_{C} ; {Mass\_Labile}_{t=0}=k_{PropLabile_{Birth}}\cdot BodyMass_{Birth}$ | E36 |
| $Mass_{Struct}$ | Structural mass (kg) | $\frac{dMass_{Struct}}{dt}=ME_{Growth}{\cdot Eff_{GainNonLabile}}/{EV_{GainNonLabile}}$ | E37 |
| $Mass_{DigCont}$ | Digestive contents (kg) | $Mass_{DigCont}=7.8+19.6\cdot\left( AcqT\cdot NDF_{Res} \right)-1.06\cdot\left( AcqT\cdot NDF_{Res} \right)^{2}-0.7*6$ | E38^d^ |
| $Mass$ | Total body mass (kg) | $Mass=Mass_{Struct}+Mass_{Labile}+Mass_{Uterus}+Mass_{DigCont}$ | E39 |
| $Milk$ | Milk production (kg/day) | $Milk=ME_{Milk}\cdot\frac{EFF_{Lac}}{EV_{Milk}}$ | E40 |
| $EV_{Mnt}$ | Energy value of maintenance (MJ/kg metabolic mass) | $EV_{Mnt}=H_{EVMnt0}+\left( H_{EVMnt1}-H_{EVMnt0} \right)\cdot\frac{t^{(H_{EVMnt2})}}{{H_{EVMnt3}}^{(H_{EVMnt2})}+t^{{(H}_{EVMnt2})}}$ | E41^e^ |
| $EV_{GainStruct}$ | Energy value for the gain of 1 kg of structural mass (MJ/kg) | $EV_{GainStruct}=1.035\cdot\left( {Mass_{Struct}}/{Mas{s_{Struct}}_{theo}} \right)+5.247$ | E42^f^ |
| $EFF_{GainStruc}$ | Efficiency of conversion ME to NE for the gain of 1 kg of structural mass | $EFF_{GainStruc}=0.0322\cdot\left( {Mass_{Struct}}/{Mass_{Struct}} \right)+0.2002$ | E43^f^ |
| $EBF$ | Empty body fat (kg) | $EBF=Mass_{Labile}\cdot0.75+Mass_{Struct}\cdot0.03\cdot{Mass_{Struct}}/{Mas{s_{Struct}}_{theo}}$ | E44^g^ |
| $BCS$ | Body condition score | $BCS=\frac{{EBF}/{\left( 1-e^{-0.0068\cdot\left( Mass_{Labile}+Mass_{Struct}-EBF \right)} \right)^{3.7235}-17.9}}{39.8}$ | E45^g^ |
| $P_{CONC}$ | Probability of conception | $P_{CONC}=0.6\cdot{P_{CONC}}_{BCS}\cdot{P_{CONC}}_{EB}\cdot{P_{CONC}}_{MY}$ | E46^h^ |
| $P_{CONC_{BCS}}$ | Modulation of probability of conception depending on BCS | ${P_{CONC}}_{BCS}=min(1 , 2\cdot1/{1+e^{-0.425\cdot\left( BCS-3 \right)}}$ | E47^h^ |
| ${P_{CONC}}_{EB}$ | Modulation of probability of conception depending on energy balance | ${P_{CONC}}_{EB}=min\left( 1 , 2\cdot\left( 1/{1+e^{-0.027\cdot-ME_{LabileMob}}} \right) \right)$ | E48^h^ |
| ${P_{CONC}}_{MY}$ | Modulation of probability of conception depending on milk production | ${P_{CONC}}_{MY}=min\left( 1 , 2\cdot\left( 1/{1+e^{0.012\cdot Milk}} \right) \right)$ | E49^h^ |
| $P_{SURV}$ | Survival probability^*^ | $\left\{ \begin{aligned} if AccMnt_{Def}\geq1.5 or Cull_{Stat}=1 at DRYING, then P_{SURV}=0 \\ else P_{SURV}=1 \end{aligned} \right.$ | E50 |
| **Boolean variables, counters and timers** | |  |  |
| $Alive_{Stat}$ | Living status of the female | $DEATH$ |  |
| $Cull_{Stat}$ | Culling status of the female | $CULLING$ |  |
| $Gest_{Stat}$ | Gestating status of the female | $CONCEPTION, PARTURITION$ |  |
| $Lac_{Stat}$ | Lactating status of the female | $PARTURITION, DRYING$ |  |
| $VarStock_{Stat}$ | Energy balance status of the female | $\left\{ \begin{aligned} if ME_{VarStock}\geq0, then VarStock_{Stat}=1 \\ else VarStock_{Stat}=0 \end{aligned} \right.$ | E51 |
| $Gest_{Nb}$ | Number of gestation | $CONCEPTION$ |  |
| $Lac_{Nb}$ | Number of lactation | $PARTURITION$ |  |
| $Gest_{Time}$ | Gestation time (days) | $CONCEPTION, PARTURITION$ |  |
| $Lac_{Time}$ | Lactation time (days) | $PARTURITION, DRYING$ |  |
| $Age_{NextOestrus}$ | Age at the next oestrus (days) | $CONCEPTION$ |  |
| $Age_{LastPart}$ | Age at last parturition (days) | $PARTURITION$ |  |

^a^Additional file 5 Section ”Calibration of the proportion of energy allocated to gestation, representing allocation for future progeny AllocPf”

^b^Additional file 5 Section ”Effects of parity on lactation acquisition”

^c^[16]

^d^[28]

^e^Additional file 5 Section ”Age dependence of the energetic value for maintenance EV_Mnt”

^f^Additional file 5 Section”Effect of the degree of maturity during growth on the energetic value of structural mass gain and growth function efficiency”

^g^[18,19]

^h^[17]
