# Supplementary material for: Disentangling the relative roles of resource acquisition and allocation on animal feed efficiency: insights from a dairy cow model
Source: Genet Sel Evol. 2016 Sep 26;48:72. doi: 10.1186/s12711-016-0251-8 (PMC5037647; doi:10.1186/s12711-016-0251-8)
Supplement: Supplementary file 3 — Additional file 3: Table S3. Description of the discrete events in the physiological sub-model. Description: The table provides the description of the triggers that activate the discrete events and the actions on model’s elements implemented by the events. [file 12711_2016_251_MOESM3_ESM.docx]

**Additional file 3 Table S3 Description of the discrete events in the physiological sub-model**

| **Name** | **Trigger** | **Actions** |
| --- | --- | --- |
| $CONCEPTION$ | $\left\{ \begin{aligned} t\geq Age_{NextOestrus} \\ P_{CONC}\geq0.4 \\ Gest_{Stat}=0 and Alive_{Stat}=1 \end{aligned} \right.$ | $if rand\left( 0,1 \right)\leq P_{CONC} then \left\{ \begin{aligned} Gest_{Stat}=1, Gest_{NB}=Gest_{Nb}+1 \\ if Gest_{Nb}>1, k_{H}Pf_{4}=0 \end{aligned} \right.$  $else Age_{NextOestrus}=t+Length_{Oestrus}$ |
| $PARTURITION$ | $\left\{ \begin{aligned} Gest_{Time}\geq Length_{Gest} \\ Gest_{Stat}=1 and Alive_{Stat}=1 \end{aligned} \right.$ | $Gest_{Stat}=0 ;Lac_{Stat}=1;Lac_{Nb}=Lac_{Nb}+1 ;Gest_{Time}=0$  $Age_{NextOetrus}=t+Lenght_{WaitPeriod}+Length_{Oestrus}$  $Age_{LastPart}=t$  $AllocS_{2}=k_{init}Pc_{4}+k_{init}Pc_{1}\cdot\frac{k_{init}Pc_{2}}{k_{init}Pc_{3}-k_{init}Pc_{2}}\cdot\left( e^{-(Gest_{Nb}\cdot k_{init}Pc_{2})}-e^{-(Gest_{Nb}\cdot k_{init_{Pc_{3}}})} \right)$  $AllocPc=AllocPf;AllocPf=0;AllocS_{1}= 1-AllocS_{2}-AllocG-AllocPc$  $Mass_{Uterus}=0$  $AcqL_{GENpctMAT}={min(k}_{M}AcqL_{1}\cdot\left( \left( {k_{M}AcqL_{2}}/\left( k_{M}AcqL_{3}-k_{M}AcqL_{2} \right) \right)\cdot\left( e^{-{(k}_{M}AcqL_{2}\cdot Gest_{Nb})}-e^{-(k_{M}AcqL_{3}\cdot Gest_{Nb})} \right)+k_{M}AcqL_{4} \right),1)$  $k_{D}AcqL_{1}=1.91+(0.03\cdot{{Gest}_{Nb}^{15}}/{{3.2}^{15})+{Gest}_{Nb}^{15}}$  $if Gest_{Nb}>1 then k_{D}AcqL_{3}=0.00185 else k_{D}AcqL_{3}=0.0016$ |
| $DRYING$ | $\left\{ \begin{aligned} Lac_{Time}\geq Length_{Lac} \\ Lac_{Stat}=1 and Alive_{Stat}=1 \end{aligned} \right.$ | $Lac_{Stat}=0;Lac_{Time}=0$  $AllocS=AllocS+ AllocPc+AllocS_{2}$  $if Cull_{Stat}=1 then P_{SURV}=0$ |
| $CULLING$ | $\left\{ \begin{aligned} t-Age\_LastPart\geq200 \\ Gest_{Stat}=0 \\ Lac_{Nb}>2 \end{aligned} \right.$ | $Cull_{Stat}=1$ |
| $DEATH$ | $P_{SURV}=0$ | $Alive_{Stat}=0$ |
