# Supplementary material for: Disentangling the relative roles of resource acquisition and allocation on animal feed efficiency: insights from a dairy cow model
Source: Genet Sel Evol. 2016 Sep 26;48:72. doi: 10.1186/s12711-016-0251-8 (PMC5037647; doi:10.1186/s12711-016-0251-8)
Supplement: Supplementary file 4 — Additional file 4. Details of model description. Description: The document provides the description of all model’s equations and detailed aspects of the discrete events functioning. Figure S1 shows an example of the flows of energy simulated by the model [15–17, 28–30]. [file 12711_2016_251_MOESM4_ESM.docx]

**Additional file 4 Details of model description**

**Model initialization**

Model initialization corresponds to birth: at $t=0$, $Alive_{Stat}=1$ and the simulation time corresponds to the female’s age (in days).

Structural mass ($Mass_{Struct})$ and labile mass ($Mass_{Labile}$) are set to:

$$Mass_{Struct}\left( t=0 \right)=\left( 1-k_{LabileBirth} \right).Mass_{Birth}$$

and $Mass_{Labile}\left( t=0 \right)=k_{LabileBirth}.Mass_{Birth}$.

Allocation compartments $AllocG$ and $AllocS$ are set to:

$AllocG\left( t=0 \right)=k_{G0}$ and $AllocS\left( t=0 \right)={1-k}_{G0}$,

where $k_{G0}$ is the initial proportion of energy allocated to growth. Allocation compartments $AllocPf$and $AllocPc$are set to zero. Total acquisition corresponds to basal acquisition and is set to $0.2\cdot AcqB_{GEN}$.

**Allocation sub-model**

The allocation sub-model is made up of four compartments reflecting priorities for four life functions: growth, future progeny, current progeny and survival. Dynamics of these compartments are based on mass action laws to represent the progressive transfers of priority among functions throughout physiological states. The rate of change in the proportion of energy allocated to growth, $AllocG$ is defined by the differential equation:

$\frac{dAllocG}{dt}=-f_{prio}G2S\cdot Alive_{Stat}.$ (1)

The flow $f_{prio}G2S$ represents the rate of decrease in allocation to growth as the female ages. It is given by:

$f_{prio}G2S=AllocG*(G2S_{GEN}+0.01*AllocPf),$ (2)

where parameter $G2S_{GEN}$ is the genetic-scaling parameter defining the rate of priority transfer from growth to somatic functions. An increase in $G2S_{GEN}$ leads to a greater priority transfer and a higher decrease of $AllocG$ level, and thus a decrease of proportion of energy allocated to growth. The priority transfer from growth to survival is further increased by $AllocPf$. Thus, when gestation starts, decrease in allocation to growth is accelerated to enable a greater priority transfer toward future progeny. It is assumed that gestation slows down growth.

The proportion of energy allocated to gestation is given by $AllocPf$, the rate of change of which is defined by the differential equation:

$\frac{dAllocPf}{dt}=+f_{prio}S2Pf\cdot Alive_{Stat}$. (3)

The flow $f_{prio}S2Pf$ represents the increase of allocation to gestation as gestation time increases. It is modelled with a rising sigmoid function that depends on gestation time and on four fixed parameters ($k_{H}Pf_{0}$, $k_{H}Pf_{1}$, $k_{H}Pf_{2}$ and $k_{H}Pf_{3}$), as currently we assumed no genetic variance for allocation to gestation [See Additional file 1 Table S1]. The proportion of energy allocated to lactation is given by $AllocPc$ defined by the differential equation:

$\frac{dAllocPc}{dt}=(+f_{prio}S2Pc-f_{prio}Pc2S)\cdot Alive_{Stat}$. (4)

The increase in the proportion of energy allocated to milk production at the beginning of lactation is described by $f_{prio}S2Pc$ as given by:

$f_{prio}S2Pc=AllocS_{2}\cdot S2P_{c}\cdot Lac\_Stat$. (5)

The parameter $S2P_{c}$drives the priority transfer from somatic functions to current progeny at the beginning of lactation. In the current version, it is fixed since no genetic variance in the priority transfer from somatic functions to current progeny is assumed.

The decrease in energy for milk production as lactation progresses is described by the flow$f_{prio}Pc2S$, given by:

$f_{prio}Pc2S=AllocPc\cdot Lac_{Stat}\cdot(Pc2S_{GEN}+AllocPf\cdot Gest_{Stat}\cdot0.06)$ (6)

The parameter $Pc2S_{GEN}$ is the genetic component for the priority transfer from current progeny to somatic functions. Changing its value affects the dynamics of $AllocPc$ and allows the representation of different strategies of lactation allocation. An increase in $Pc2S_{GEN}$ accelerates the rate of priority transfer from lactation to survival $f_{prio}Pc2S$ and thus decreases the level of allocation to lactation. This priority flow is also affected by the allocation level to future progeny. As gestation progresses, $AllocPf$ increases and accelerates the return of priority from $AllocPc$ to $AllocS$. This effect accounts for the depressive effect of gestation on lactation [15]. Finally, the proportion of energy allocated to somatic functions is given by $AllocS$ defined by the differential equation:

$\frac{dAllocS}{dt}=(f_{prio}G2S-f_{prio}S2Pf-f\_prioS2Pc+f\_prioPc2S)\cdot Alive_{Stat}$. (7)

Since there is no loss of the dimensionless quantity of one in the compartment network, at each time step the sum of compartment’s levels is equal to 1, ensuring energy conservation.

**Acquisition sub-model**

The acquisition sub-model simulates the quantity of DM acquired from the nutritional environment, depending on resource characteristics (gross energy density, fibre content and proportion of concentrate feedstuff), and its conversion into ME through digestion.

The total daily intake of DM, is given by:

$AcqT=AcqB+AcqL$. (8)

The variable $AcqB$ is defined by:

$AcqB=(AcqB_{GEN}-0.8\cdot AcqB_{GEN} \cdot e^{-k_{AcqB_{MAT}}\cdot t})\cdot Alive_{Stat}.$ (9)

The parameter $AcqB_{GEN}$ is the genetic-scaling parameter driving basal acquisition. It represents the asymptote of the curve, corresponding to the maximum intake of DM at maturity for a non-lactating animal. Although intake is frequently expressed as a percentage of body weight, we explicitly chose not to do this. It would create an a priori correlation between allocation to growth and acquisition and prevents to study the relative roles of these components on the phenotypic traits and FE. The variable $AcqL$ is defined by:

$AcqL=Lac_{Stat}\cdot Alive_{Stat}\cdot AcqL_{Max}\cdot AcqL_{Dyn}$. (10)

The shape of the curve during lactation is given by the $AcqL_{Dyn}$ component of Equation (E15) in Table S2 [See Additional file 2 Table S2]. The maximum DMI reached during lactation is given by $AcqL_{Max}$, which depends on the genetic-scaling parameter$AcqL_{GEN}$. This latter is the maximum DM reached at maturity to account for a maturation of the potential to acquire resource as female ages (Equation E14 in Table S2 [see Additional file 2 Table S2]. The total intake of DM is converted into ME available for allocation,$ME_{Acq}$ depending on the energy density of the DM available in the environment, $GE_{Res}$ in Mcal/kg, and the metabolizability of the diet, $ME_{PctGE}$, as given by:

$ME_{Acq}= (AcqB+AcqL)\cdot GE_{Res}\cdot ME_{PctGE}$. (11)

Diet metabolizability accounts for the energy losses through feces, urine and enteric methane during digestion. It is affected by the level of dry matter intake and the proportion of concentrate as proposed by [16].

Finally, the acquisition sub-model also determined the digestive contents, $Mass_{DigCont}$ depending on DM intake and resource fiber content [28] and given by:

$$Mass_{DigCont}=$$

$7.8+19.6\cdot\left( Acq\cdot NDF_{PropDMI} \right)-1.06\cdot\left( Acq\cdot NDF_{PropDMI} \right)^{2}-0.7*6$. (12)

This variable is taken into account in the total mass of the female, contributing to its maintenance costs. This choice was made because we assumed that a high capacity of acquiring resource has some energy costs (development and maintenance of digestive structures for instance). We incorporated these costs by indexing maintenance requirements on the total body mass and therefore taking into account digestive contents.

**Energy utilization sub-model**

The energy utilization sub-model combines ME from the acquisition sub-model with partition coefficients from the allocation sub-model and simulates the conversion of energy allocated to physiological functions (growth, gestation, lactation and somatic functions) using the following equations:

$ME_{Growth}= AllocG\cdot ME_{Acq}$ (13)

$ME_{GravidUterus}=AllocPf\cdot ME_{Acq}$ (14)

$ME_{Milk}=AllocPc\cdot ME_{Acq}$ (15)

$ME_{Soma}=AllocS\cdot ME_{Acq}$ (16)

The energy allocated to somatic functions is assumed to be first used for maintenance, calculated as follows:

$ME_{MntReq}=\frac{EV_{Mnt}\cdot{BodyMass}^{0.75}}{EFF_{Mnt}}$. (17)

The difference between the level of requirements and energy allocated to somatic functions determines the change in energy storage$ME_{VarStock}$, as given by:

$ME_{VarStock}=ME_{Soma}-ME_{MntReq}$. (18)

The sign of the state $ME_{VarStock}$ determines whether female’s reserves are mobilized or reconstituted. This state corresponds to a Boolean variable $k_{Stat}$. If $ME_{Soma}$ is above$ME_{MntReq}$, the excess is stored in labile mass as follows:

$ME_{LabileRep}=ME_{VarStock}\cdot VarStock_{Stat}$. (19)

If $ME_{Soma}$ is below$ME_{MntReq}$, labile mass is mobilized to cover the difference as follows:

$ME_{LabileMob}=min\left( \left( \left( 1-VarStock_{Stat} \right)\cdot-ME_{VarStock} \right),(Mass_{Labile}\cdot EV_{LossLabile}\cdot Eff_{LossLabile}) \right)$. (20)

The energy for maintenance is given by:

$ME_{Mnt}=ME_{Soma}-ME_{LabileRep}+ME_{LabileMob}$. (21)

Mobilization of reserves may not cover the difference between $ME_{MntReq}$and$ME_{Soma}$ leading to a deficit in energy for maintenance given by:

$ME_{MntDeficit}=(ME_{Mnt}-ME_{MntReq})/ME_{MntReq}$. (22)

Daily deficits in covering maintenance requirements are accumulated in$Acc_{MntDef}$. When $Acc_{MntDef}$ exceeds 1.5, $P_{SURV}=0$. This threshold represents around 15 days during which maintenance requirements are not covered.

Senescence can be simulated by conditioning mortality to a deficit in maintenance. In this case energetic value to maintain 1 kg of metabolic weight ($EV_{Mnt}$) is made age-dependent using the following equation:

$EV_{Mnt}=H_{EVMnt0}+\left( H_{EVMnt1}-H_{EVMnt0} \right)\cdot\frac{t^{H_{EVMnt2}}}{\left( {H_{EVMnt3}}^{H_{EVMnt2}} \right)+(t^{H_{EVMnt2}})}$ (23)

This equation reflects an increase of maintenance costs with age (Equation E41 in Table S2 [See Additional file 2Table S2]) as proposed by [29] to represent an increase in energy expenditure with age as reparative costs due to oxidative damage become higher. Increase in maintenance costs leads to an increasing use of reserves to cover requirements until the whole labile mass is used. Then the deficit in covering maintenance requirements increases, leading to death.

The quantities of energy allocated to functions are presented in Figure S1 [See Additional file 4 Figure S1]. These quantities are converted into matter (body mass components and milk) with coefficients that represent the energetic costs of 1 kg of matter and efficiency coefficients to account for losses when converting metabolizable energy into net energy.

Body mass is decomposed into 4 compartments as given by:

$Mass=Mass_{Struct}+Mass_{Labile}+Mass_{Uterus}+Mass_{DigCont}$ (24)

The structural mass compartment $Mass_{Struct}$ represents the part of body mass that cannot be mobilized and that is fuelled by growth as defined by the differential equation:

$\frac{dMass_{Struct}}{dt}=ME_{Growth}{\cdot Eff_{GainNonLabile}}/{EV_{GainNonLabile}}$. (25)

The labile mass compartment $Mass_{Labile}$ represents the energetic reserves of the female that vary throughout life depending on the balance between anabolic and catabolic processes, as given by:

$\frac{dMass_{Labile}}{dt}=Labile_{A}-Labile_{C}$. (26)

The flow $Labile_{A}$ representing anabolism is given by:

$Labile_{A}={ME_{VarStock}\cdot VarStock_{Stat}\cdot EFF_{GainLabile}}/{EV_{GainLabile}}$. (27)

The flow $Labile_{C}$ representing catabolism is given by:

$$Labile_{C}=$$

$min\left( \left( 1-VarStock_{Stat} \right)-ME_{VarStock}\cdot\frac{1}{EFF_{LossLabile}}\cdot\frac{1}{EV_{LossLabile}},Mass_{Labile} \right)$ (28)

The uterus compartment, $Mass_{Uterus}$ accounts for foetal and uterus growth during gestation. It is defined by:

$\frac{dMass_{Uterus}}{dt}=AllocPf\cdot ME_{Acq}\cdot Gest_{Stat}\cdot\frac{1}{EV_{GainUterus}}$. (29)

The energy allocated to lactation is converted into milk as given by:

$Milk=ME_{Milk}\cdot\frac{EFF_{Lac}}{EV_{Milk}}$. (30)

Milk composition stays constant over the whole lactation and thus the model does not allow changes in efficiency due to changes in milk composition. Finally, the utilization sub-model simulates the probability of conception, $P_{CONC}$, which is used in the $CONCEPTION$event as proposed by [17]. It is defined as follows:

$P_{CONC}=0.6\cdot{P_{CONC}}_{BCS}\cdot{P_{CONC}}_{EB}\cdot{P_{CONC}}_{MY}$. (31)

The likelihood of conception is set at a basal level of 0.6, which corresponds to the probability of conception of a virgin heifer [30]. This basal value is modulated by three factors:$P_{CONC_{MY}}$ ${P_{CONC}}_{BCS}$ and${P_{CONC}}_{EB}$. Milk production affects$P_{CONC_{MY}}$to account for the negative effect of milk production on fertility. ${P_{CONC}}_{BCS}$depends on $BCS$to account for the effect of the actual level of reserves on fertility. ${P_{CONC}}_{EB}$ depends on energy balance, defined by to account for the dynamic effect of reserves utilization on fertility. The equations E46, E47, E48, E49 and E50 are in Table S2 [See Additional file 2 Table S2].

**Physiological status sub-model**

The physiological status sub-model simulates the changes in female physiological status. The changes can be triggered by time-dependent biological function (e.g. the parturition event occurs 280 days after conception). The changes can also be triggered by management decisions (e.g. culling). It is made up of five discrete events, which are summarized in Table S3 [see Additional file 3 Table S3]: $CONCEPTION$,$PARTURITION$,$DRYING$, $CULLING$, and$DEATH$. These events drive change in Boolean variables reflecting whether the female is alive or not ($Alive_{Stat}$), culled or not ($Cull_{Stat}$), gestating or not ($Gest_{Stat}$) and lactating or not ($Lac_{Stat}$). These events also drive physiological timing (in days) related to gestation ($Gest_{Time}$), and lactation ($Lac_{Time}$), and they update pregnancy and lactation numbers ($Gest_{Nb}$ and$Lac_{Nb}$).

The $CONCEPTION$event operates the potential change from non-pregnant to pregnant status if fertilization occurs. The $CONCEPTION$ event is triggered if the female is in an oestrus period ($t=Age_{Oestrus}$) and if the probability of conception ($P_{CONC}$) is above 0.4. This rule corresponds to the assumption that below this value, the female does not exhibit oestrus and thus cannot be fertilized [17].

The fertilization process is simulated using a random draw from a uniform distribution, U (0, 1). If the value is smaller than the value of $P_{CONC}$, the female is assumed to be pregnant and $Gest_{Stat}$ switches from 0 to 1. As a result, the priority flow for future progeny ($f_{prio}S2Pf$) is activated, energy is used for gestation and the gravid uterus grows. Losses due to early embryo death were included in $P_{CONC}$ calculation. If the random draw is greater than$P_{CONC}$, the female is not pregnant and the next oestrus occurs after $Length_{Oestrus}$ days.

The $PARTURITION$ event is triggered after duration of $Length_{Gest}$ days. It updates Boolean variables ($Gest_{Stat}$ from 1 to 0 and $Lac_{Stat}$ from 0 to 1) and gestation and lactation numbers ($Gest_{Nb}$ and$Lac_{Nb}$). It also causes a discrete transfer among allocation and mass compartments [See Additional file 3 Table S3] and updates the calculated parameters to account for parity effect on the lactation acquisition curve [See Additional file 3 Table S3].

The$CULLING$ event tests every day if the female is to be culled or not. If yes, $Cull_{Stat}$ switches to 1. The event $DRYING$ is triggered after duration of $Length_{Lac}$ days. It updates lactating status ($Lac_{Stat}$ from 1 to 0) and causes the discrete transfer of priority between $AllocPc$ and$AllocS$. It also updates $P_{SURV}$ depending on $Cull_{Stat}$.

Finally, the event $DEATH$ is triggered if $P_{SURV}=0$. It stops simulation by switching the Boolean variable $Alive_{Stat}$from 1 to 0. In this version of the model, the event $DEATH$ occurs if the female is culled ($Cull_{Stat}=1$) or if the female accumulates daily deficits in covering maintenance requirements ($Acc_{MntDef})\geq1.5$.


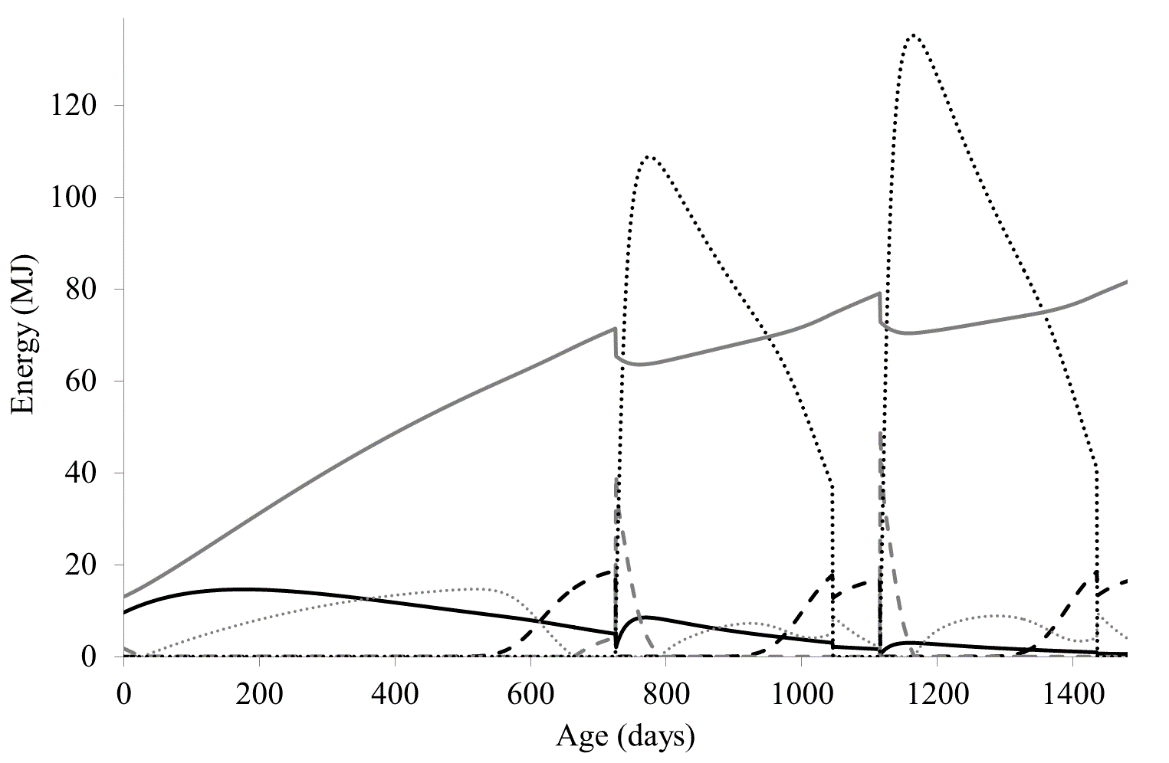


**Figure S1. Dynamic changes in quantities of energy simulated by the utilization sub-model throughout two reproductive cycles of a dairy cow.**

Based on the metabolizable energy (ME in J) acquired simulated by the acquisition sub-model and the partition coefficients simulated by the allocation sub-model, the utilization sub-model simulates ME for growth (black solid line), ME for gestation (black dashed line), ME for lactation (black dotted line), ME for maintenance (grey solid line), ME mobilized from body reserves (grey dashed line and ME for body reserves repletion (grey dotted line).
