# Supplementary material for: Disentangling the relative roles of resource acquisition and allocation on animal feed efficiency: insights from a dairy cow model
Source: Genet Sel Evol. 2016 Sep 26;48:72. doi: 10.1186/s12711-016-0251-8 (PMC5037647; doi:10.1186/s12711-016-0251-8)
Supplement: Supplementary file 5 — Additional file 5. Details of the calibration of the model. The document provides a description of some detailed aspects of the model’s calibration. It indicates the assumptions that were made to introduce biological relationships in the model and how the values of the parameters for these relationships were defined. The relationships relate to the effect of the degree of maturity on the energetic value of structural mass gain and growth function efficiency, the proportion of energy allocated to gestation (Figure S2), the age-dependence of the energetic value for maintenance (Figures S3, S4 and S5), the effect of parity on lactation acquisition (Figures S6, S7 and S8) and the effect of parity on lactation allocation (Figure S9) [18, 19, 31, 32]. [file 12711_2016_251_MOESM5_ESM.docx]

**Additional file 5 Details of model calibration**

**Effect of the degree of maturity during growth on the energetic value of structural mass gain and growth function efficiency**

During growth, composition of structural mass gain varies due to allometric development of tissues. Roughly, more proteins are accreted during early stage of life and more fat in late stage of life. Regarding the model presented here, composition of structural mass gain determines the energetic value of 1 kg of structural mass and the efficiency of the growth function in converting metabolizable energy (ME) into net energy (NE). Indeed, 1 kg of fat contains more net energy than 1 kg of proteins (39.762 MJ/kg and 23.857 MJ/kg [31]. In addition, partial net efficiencies of metabolizable energy utilization for protein and lipid gain are respectively 0.20 and 0.75 [32]. Most of the models accounting for growth used a degree of maturity to drive gain composition. This degree of maturity is easy to obtain since it is generally defined as the ratio between body mass at a given time *t* and body mass at maturity, with this latter being a model input. In our model, body mass at maturity is not an input. It is the result of acquisition and allocation trajectories, as given by:

$$\frac{dMass_{Struct}}{dt}=ME_{Growth}{\cdot Eff_{GainStruct}}/{EV_{GainStruct}}$$

with $ME_{Growth}=AllocG\cdot ME_{acq}$.

In order to account for change in the composition of gain during growth, we need to approximate body mass at maturity. We defined $Mas{s_{Struct}}_{theo}$as the structural mass at maturity. It corresponds to$\left( \lim_{\infty} Mass_{Struct} \right)$ with a fixed resource energy density (21.275 MJ/kg of DM) and metabolizability (0.578 MJ NE/MJ ME). Variation in $Mass_{Struct}$is given by:

$\frac{dMass_{Struct}}{dt}=AllocG\cdot ME_{acq}\cdot EFF_{GainStruct}/EV_{GainStruct}$.

The algebraic form of $AllocG$is given by:

$AllocG\left( t \right)=G_{0}\cdot e^{-G2S_{POT}\cdot t}$.

Variation of $Mass_{Struct}$ is thus:

$\frac{dMass_{Struct}}{dt}=(G_{0}\cdot e^{-G2S_{GEN}\cdot t})\cdot(AcqB_{GEN}-0.8\cdot AcqB_{GEN}e^{-kAcqB_{MAT}\cdot t})\cdot G{E_{Res}}_{Ref}\cdot ME_{pctGERef}\cdot{EFF_{GainStruct}}/{EV_{GainStruct}}$.

We define ${C_{MassStruct}}_{theo}$as:

${C_{MassStruct}}_{theo}=G_{0}\cdot G{E_{Res}}_{Ref}\cdot ME_{pctGEref}\cdot{EFF_{GainStruct}}/{EV_{GainStruct}}$.

Variation of $Mass_{Struct}$ is thus:

$$\frac{dMass_{Struct}}{dt}={C_{MassStruct}}_{theo}\cdot e^{-G2S_{GEN}\cdot t}\cdot(AcqB_{GEN}-0.8\cdot AcqB_{GEN}e^{-kAcqB_{MAT}\cdot t})$$

$\leftrightarrow\frac{dMass_{Struct}}{dt}={C_{MassStruct}}_{theo}\cdot{AcqB_{GEN} \cdot e}^{-G2S_{GEN}\cdot t}-{C_{MassStruct}}_{theo}\cdot AcqB_{GEN}\cdot0.8\cdot e^{-(G2S_{GEN}+kAcqB_{MAT})\cdot t})$

$\leftrightarrow\frac{dMass_{Struct}}{dt}={C_{MassStruct}}_{theo}\cdot{AcqB_{GEN} \cdot(e}^{-G2S_{GEN}\cdot t}-0.8\cdot e^{-(G2S_{GEN}+kAcqB_{MAT})\cdot t})$.

The algebraic form $Mass_{Struct}(t)$ is given by:

$\int_{0}^{\infty} {C_{MassStruct}}_{theo}\cdot{AcqB_{GEN} \cdot(e}^{-G2S_{GEN}\cdot t}-0.8\cdot e^{-(G2S_{GEN}+kAcqB_{MAT})\cdot t})+Mass_{Struct}\left( t=0 \right)$.

We assume that $EFF_{GainStruct}/EV_{GainStruct}$ is constant and equal to $ratio_{GainStruct}$. This assumption is based on GARUNS equations [18, 19]. We computed energetic value and efficiency related to growth based on these equations. The ratio variation along gradient of maturity from 0 to 1 was 1.03%. We choose the value 0.037544089 for $ratio_{GainStruct}$corresponding to maturity of 0.5.

The initial value of $Mass_{Struct}$is given by $Mass_{Birth}\cdot{{(1-k}_{Labile}}_{Birth})$ which are model inputs.

Therefore we can write:

$\leftrightarrow Mass_{Struct}\left( t \right)={C_{MassStruct}}_{theo}\cdot AcqB_{GEN}\cdot\left( \int_{birth}^{maturity} e^{-G2S_{GEN}\cdot t}\cdot dt-0.8\cdot\int_{birth}^{maturity} e^{-(G2S_{GEN}+k_{AcqB_{MAT}})\cdot t}\cdot dt \right)+Mass_{Struct}\left( t=0 \right)$,

$\leftrightarrow Mass_{Struct}\left( t \right)={C_{MassStruct}}_{theo}\cdot AcqB_{GEN}\cdot((\frac{-1}{G2S_{GEN}}\cdot e^{-G2S_{GEN}\cdot t}+C1)-0.8\cdot(\frac{-1}{G2S_{GEN}+{k_{AcqB}}_{MAT}}\cdot e^{-\left( G2S_{GEN}+k_{AcqB_{MAT}} \right)\cdot t}+C2))+Mass_{Birth}\cdot{{(1-k}_{Labile}}_{Birth})$,

with *C1* and *C2* being integration constants.

The theoretical structural mass,$Mas{s_{Struct}}_{theo}$, corresponds to the asymptotic value of $Mass_{Struct}$. It is given by the positive infinity limit of $Mass_{Struct}\left( t \right)$ and is equal to:

$Mas{s_{Struct}}_{theo}={C_{MassStruct}}_{theo}\cdot AcqB_{GEN}\cdot\left( C1-0.8\cdot C2 \right)+Mass_{Birth}\cdot{{(1-k}_{Labile}}_{Birth})$.

We need to determine$\left( C1-0.8\cdot C2 \right)$.

At birth, $Mass_{Struct}\left( t=0 \right)=Mass_{Birth}\cdot{{(1-k}_{Labile}}_{Birth})$

Therefore

${C_{MassStruct}}_{theo}\cdot AcqB_{GEN}\cdot\left( \frac{-1}{G2S_{GEN}}\cdot e^{-G2S_{GEN}\cdot t}+C1-0.8\cdot\left( \frac{-1}{G2S_{GEN}+{k_{AcqB}}_{Mat}}\cdot e^{-\left( G2S_{GEN}+k_{AcqB_{MAT}} \right)\cdot t}+C2 \right) \right)=0$,

${{\leftrightarrow C}_{MassStruct}}_{theo}\cdot AcqB_{GEN}\cdot\left( \frac{-1}{G2S_{GEN}}+C1-0.8\cdot\left( \frac{-1}{G2S_{GEN}+{k_{AcqB}}_{MAT}}+C2 \right) \right)=0$,

$\leftrightarrow\frac{-1}{G2S_{GEN}}+C1-0.8\cdot\left( \frac{-1}{G2S_{GEN}+{k_{AcqB}}_{MAT}}+C2 \right)=0$,

$\leftrightarrow C1-0.8\cdot C2=\frac{1}{G2S_{GEN}}-0.8\cdot\frac{1}{G2S_{GEN}+{k_{AcqB}}_{MAT}}$.

By using this expression in $Mas{s_{Struct}}_{theo}$equation, we obtain:

$Mas{s_{Struct}}_{theo}={C_{MassStruct}}_{theo}\cdot AcqB_{GEN}\cdot\left( \frac{1}{G2S_{GEN}}-0.8\cdot\frac{1}{G2S_{GEN}+{k_{AcqB}}_{MAT}} \right)+Mass_{Birth}\cdot{{(1-k}_{Labile}}_{Birth})$.

This theoretical value of structural mass asymptote is used to assess the degree of maturity of the simulated female. The equations used in the GARUNS model [20, 19] are based on change in fat and protein contents depending on maturity. This change in chemical composition leads to change in energetic value and efficiency. Based on these equations, we directly compute the energetic value (MJ) of 1kg of structural mass, $EV_{GainStruct}$ and the efficiency for conversion of ME into NE, $EFF_{GainStruct}$depending on maturity, as given by:

$EV_{GainStruct}=1.035\cdot({Mass_{Struct}}/{Mas{s_{Struct}}_{theo})+5.247}$.

$E{FF}_{GainStruct}=0.0322\cdot({Mass_{Struct}}/{Mas{s_{Struct}}_{theo})+0.2002}$.

**Calibration of the proportion of energy allocated to gestation, representing allocation for future progeny** $\boldsymbol{AllocPf}$

The compartment $AllocPf$ represents the dynamic change in the proportion of energy allocated to gestation. The compartment dynamics depends on the flow $f_{prio}S2Pf$ as given by:

$\frac{dAllocPf}{dt}=+f_{prio}S2Pf\cdot Alive_{Stat}$.

To determine the mathematical formalism of this flow, we used the simulations of the GARUNS model [18,19] resulting from the calibration on compilation of data from the literature. This model generates the dynamic change of energy for gestation and energy intake. We then calculate the corresponding proportion of energy allocated to gestation. As shown in Figure S2, the shape is close to a Hill function. We adjusted parameters of a Hill equation on the GARUNS curve and then we derived the Hill function to obtain the equation for the flow.


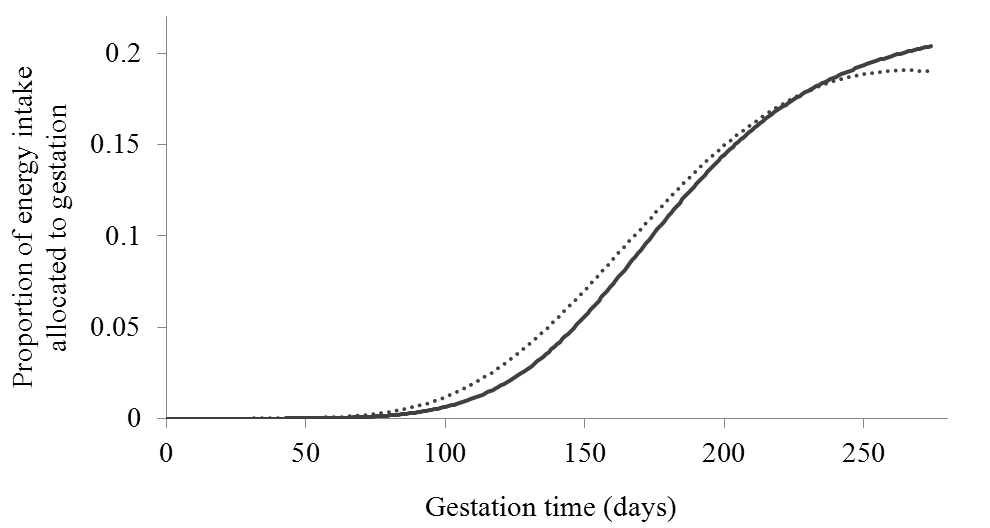


**Figure S2. Comparison of the dynamic change in the proportion of energy allocated to gestation for the GARUNS model (dotted line) and the model (solid line).**

**Age dependence of the energetic value for maintenance** $\boldsymbol{E}\boldsymbol{V}_{\boldsymbol{Mnt}}$

The maintenance requirements, $E_{MntReq}$ , are given by:

$ME_{MntReq}=EV_{Mnt}\cdot{{Mass}^{0.75}}/{EFF_{Mnt}}$.

The parameter $EV_{Mnt}$is made age-dependent as given by:

$EV_{Mnt}=H_{EVMnt0}+\left( H_{EVMnt1}-H_{EVMnt0} \right)\cdot\frac{t^{H_{EVMnt2}}}{{H_{EVMnt3}}^{H_{EVMnt2}}+t^{H_{EVMnt2}}}$.

As shown in Figure S3, the energetic cost of maintaining 1 kg of metabolic mass increases as the female’s ages.


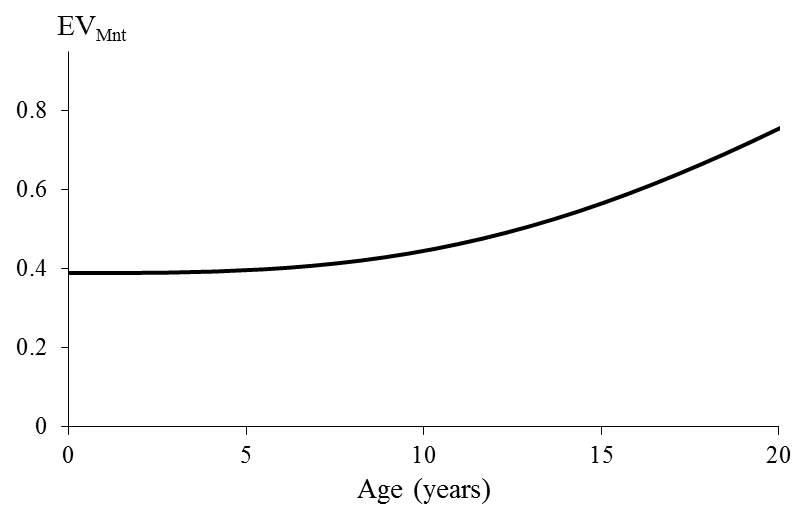


**Figure S3 Relation between female’s age and the value of the energetic costs of maintaining 1 kg of metabolic mass (**$\boldsymbol{E}\boldsymbol{V}_{\boldsymbol{Mnt}}$ **in MJ/kg^0.75^).**

This relation allows representing senescence and natural death in the model. With age, the simulated female has to spend more and more energy to maintain its mass. The labile mass is first used to sustain maintenance. Then, when there is no more labile mass, the female does not cover its requirements and starts cumulating maintenance deficit, $Acc_{MntDef}$. When the threshold of 1.5 is reached, probability of survival $P_{SURV}=0$, triggering the event $DEATH$and the simulation stops. For a female that does not reproduce and with parameters set to calibration level, the parameterization of the relation between age and $EV_{Mnt}$gives a natural death at 6503 days, almost 18 years. As shown in Figure S4, this corresponds to the age at which no more labile mass is available.


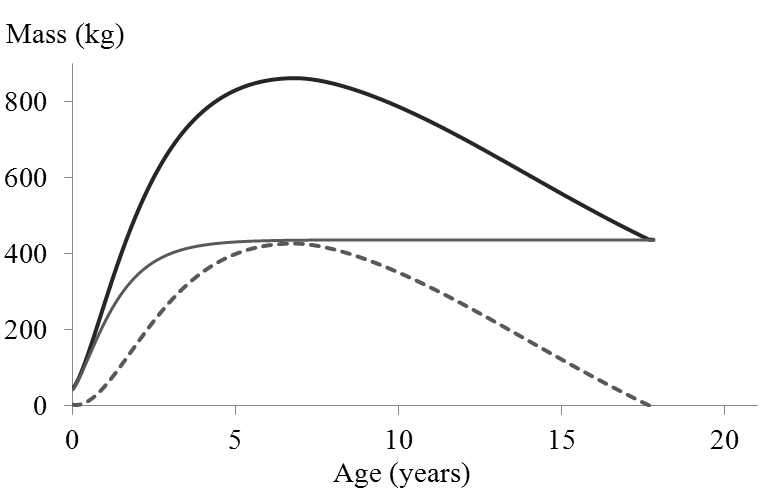


**Figure S4 Illustration of body mass trajectories simulated for a female that doesn’t reproduce and naturally dies at 6503 days of age.**

When considering reproductive cycles, age of death varies as reproduction is represented by a stochastic process. We evaluated age of death for 100 repeated simulations of calibration parameterization and with no culling. Natural death occurred at 16 years (±1.4). The mass trajectories are illustrated in Figure S5 for 10 females from the 100 repeated simulations. Variability of trajectories increases as females are ageing and reproduction is more irregular. This is due to the effect of increasing maintenance costs which implies an increase use of labile mass. As a consequence, the probability of conception is affected leading to less regular reproductive cycles.


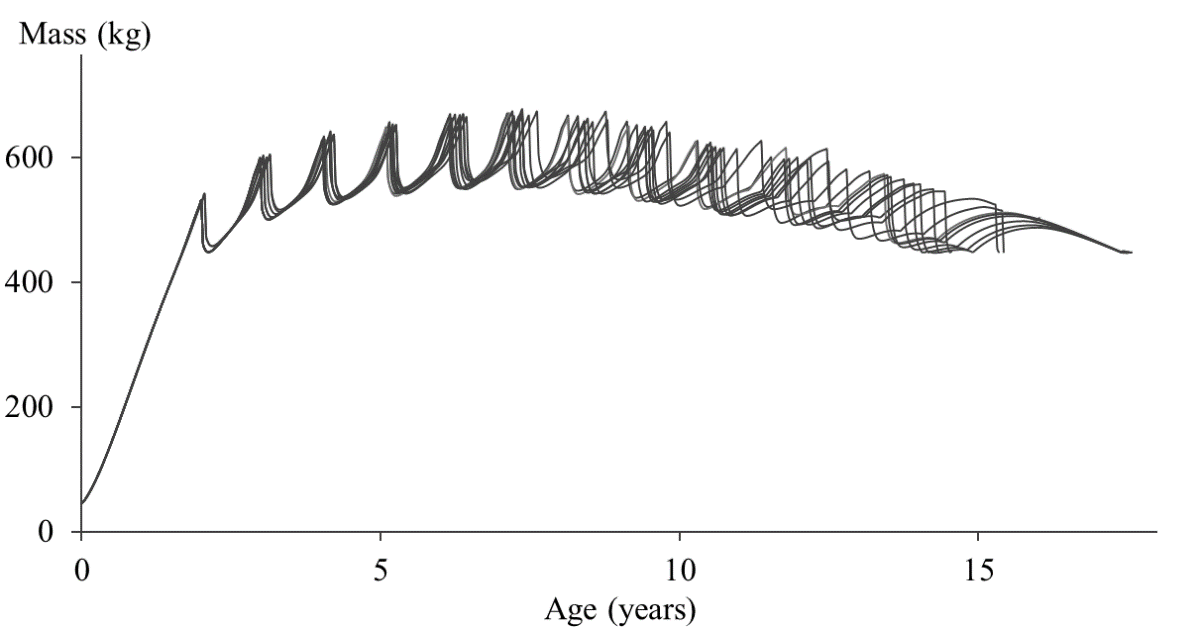


**Figure S5 Illustration of empty body mass trajectories simulated for 10 females corresponding to calibration parameterization and with no culling event.**

By manipulating the parameters of the equation for $EV_{Mnt}$it is possible to simulate different

**Effects of parity on lactation acquisition**

Acquisition of the simulated female, in kg of dry matter (DM) per day, during its lactation is defined by:

$AcqL\left( t \right)=Lac_{Stat}\cdot Alive_{Stat}\cdot AcqL_{Max}\cdot AcqL_{Dyn}(Lac_{Time})$.

The variable $AcqL_{Max}$defines the maximum dry matter intake reached during lactation. It depends on the female genetic potential and a proportion of this maximum, to account for a maturation of lactation acquisition as given by:

$AcqL_{Max}=AcqL_{GEN}\cdot AcqL_{GENpctMAT}$.

The maturation of lactation acquisition depends on female’s parity and is given by:

$AcqL_{GENpctMAT}={min(k}_{M}AcqL_{1}\cdot\left( \left( {k_{M}AcqL_{2}}/\left( k_{M}AcqL_{3}-k_{M}AcqL_{2} \right) \right)\cdot\left( e^{-k_{M}AcqL_{2}\cdot Gest_{Nb}}-e^{-k_{M}AcqL_{3}\cdot Gest_{Nb}} \right)+k_{M}AcqL_{4} \right),1)$.

With the calibration parameterization, the change in proportion of $AcqL_{GEN}$ with parity is illustrated in Figure S6.


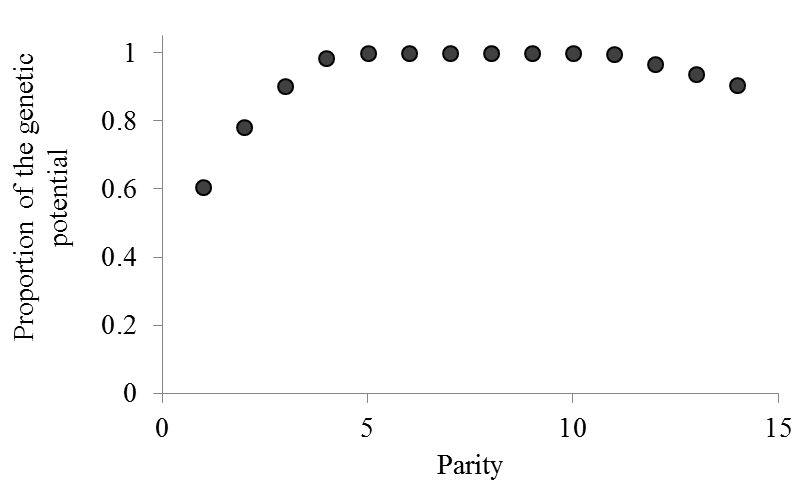


**Figure S6 Change in the proportion of genetic potential for lactation acquisition reached during lactation,** $\boldsymbol{Acq}\boldsymbol{L}_{\boldsymbol{GENpctMAT}}$ **depending on parity**

The shape of acquisition lactation trajectory is given by:

$AcqL_{Dyn}\left( Lac_{Time} \right)=\left( \left( k_{D}AcqL_{1}\cdot\frac{k_{D}AcqL_{2}}{k_{D}AcqL_{3}-k_{D}AcqL_{2}}\cdot\left( e^{-Lac_{Time}\cdot k_{D}AcqL_{2}}-e^{-Lac_{Time}\cdot k_{D}AcqL_{3}} \right) \right)-k_{D}AcqL_{4} \right)$.

This equation gives the shape of the dynamic change of acquisition during lactation and is further scaled by $AcqL_{Max}$. Therefore, values of $AcqL_{Dyn}$ are comprised between -1 and 1 as illustrated in Figure S7. At the beginning of lactation, $AcqL_{Dyn}$is negative as we considered that the first week after parturition female’s acquisition dropped. The negative value of $AcqL$ decreases the value of $AcqT$.


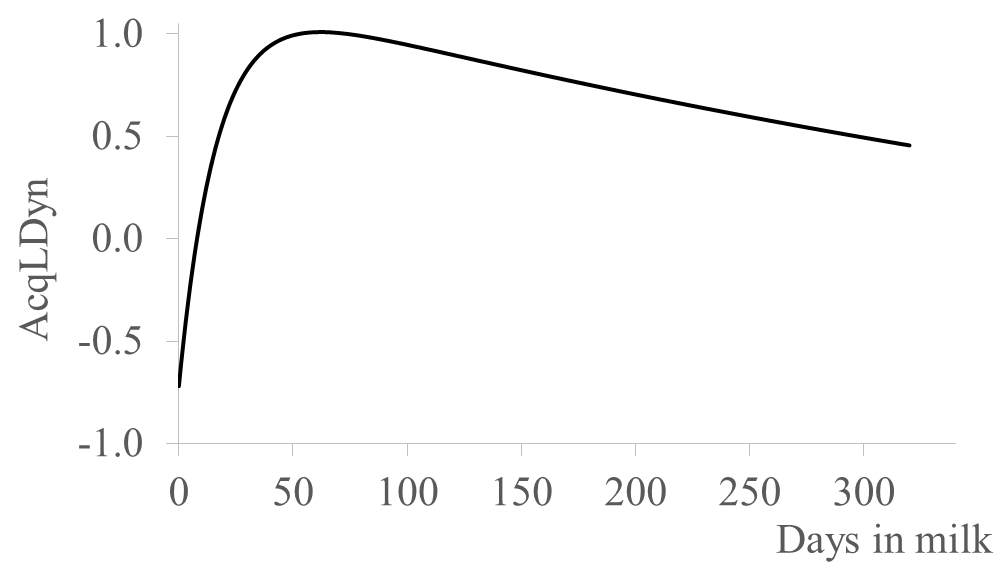


**Figure S7 Change in the shape of lactation acquisition,** $\boldsymbol{Acq}\boldsymbol{L}_{\boldsymbol{Dyn}}$**during lactation**

The parameters $k_{D}AcqL_{2}$ and $k_{D}AcqL_{4}$ are made parity dependent to represent differences in the shape of acquisition curve, based on the GARUNS data used for calibration. The differences in lactation acquisition trajectory during lactation are illustrated in Figure S8.


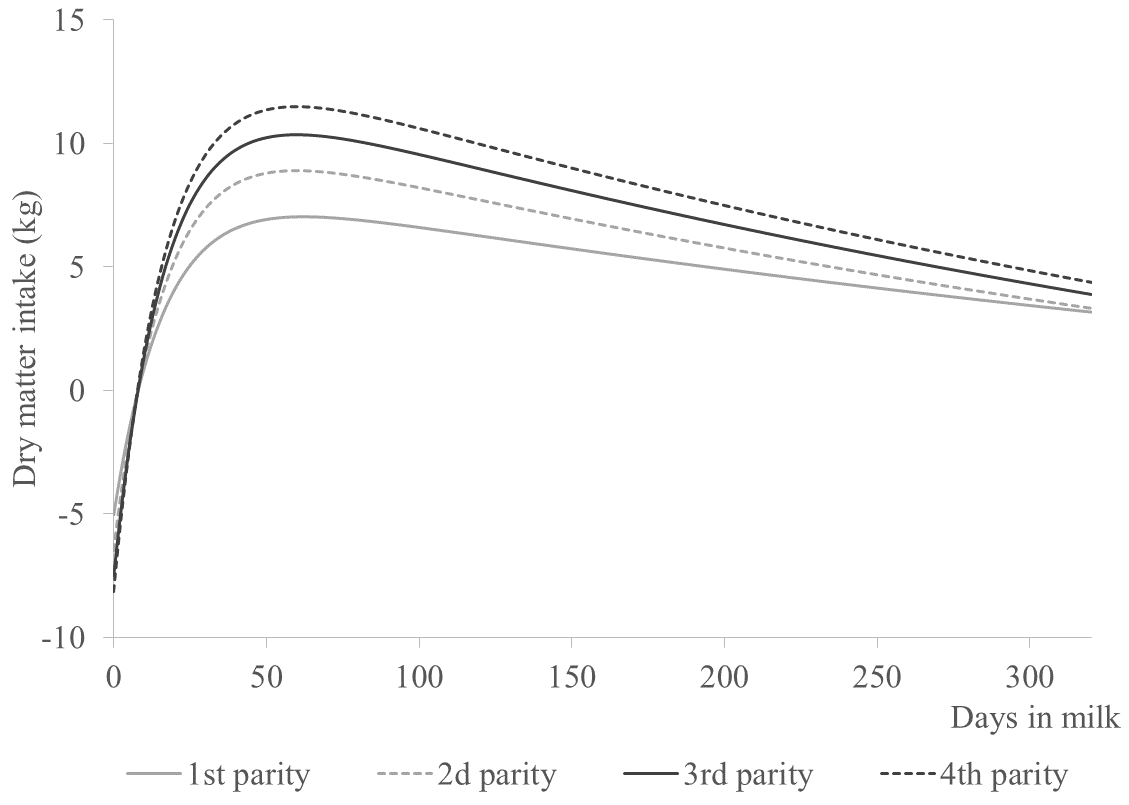


**Figure S8 Lactation acquisition trajectories for the first 4 parity of dairy cow.**

**Effects on parity on lactation allocation**

The compartment $AllocPc$ stands for the priority for current progeny and its level represents the proportion of energy allocated to lactation. The dynamic change of $AllocPc$ is defined by two flows, $f_{prio}S2PC$and $f_{prio}Pc2S$, based on mass action laws. The maximum reached by $AllocPc$ during lactation depends of flows parameters and on the initial value of $S_{2}$. The compartment $AllocS_{2}$ is a subpart of the compartment$AllocS$, made to control the transfer of priority from soma to current progeny. By decomposing $AllocS$ in $AllocS_{1}$and $S_{2}$, it is possible to manage the initial investment to current progeny independently from the return of priority for female survival as lactation progresses. We consider that the maximum proportion of energy allocated during lactation is age-dependent to account for a maturation of the female capacity to produce milk. To manipulate the maximum value of $S_{2}$, we change the initial charge of $AllocS_{2}$when the event $PARTURITION$occurs, as given by:

$AllocS_{2}(t_{Parturition}=k_{init}Pc_{4}+k_{init}Pc_{1}\cdot\frac{k_{init}Pc_{2}}{k_{init}Pc_{3}-k_{init}Pc_{2}}\cdot\left( e^{-Gest_{Nb}\cdot k_{init}Pc_{2}}-e^{-Gest_{Nb}\cdot k\_init\_Pc_{3}} \right)$.

The value changes depending on the parity and affects the maximum value of $AllocPc$ as shown in Figure S9.


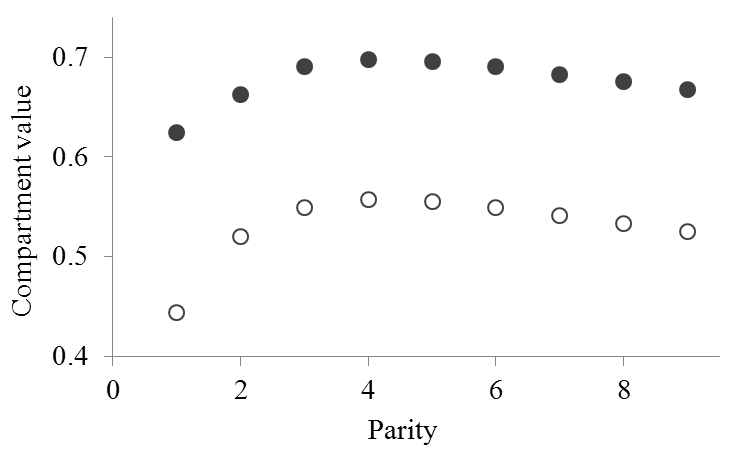


**Figure S9 Change in the value of** $\boldsymbol{Alloc}\boldsymbol{S}_{\boldsymbol{2}}$ **at parturition and in the maximal value reached by** $\boldsymbol{AllocPc}$ **during lactation, corresponding to maximal proportion of energy allocated to lactation, depending on female’s parity.**
