# Supplementary material for: Disentangling the relative roles of resource acquisition and allocation on animal feed efficiency: insights from a dairy cow model
Source: Genet Sel Evol. 2016 Sep 26;48:72. doi: 10.1186/s12711-016-0251-8 (PMC5037647; doi:10.1186/s12711-016-0251-8)
Supplement: Supplementary file 6 — Additional file 6. Calibration results and sensitivity analysis details. The document provides the results of the calibration step in the model development. This step was based on a comparison with the GARUNS model and completed by a comparison with a compilation of data from literature related to body mass (Figure S10), milk production (Figure S11) and body condition score (Figure S12). The document also provides additional information on the global sensitivity analysis with the values of the parameters used in the complete factorial design (Table S4) and on how these values of the parameters were determined. Finally, the document provides additional results of the sensitivity analysis with the variance decomposition of the two feed efficiencies depending on the input parameters (Table S5) and series of figures that show the effects of parameters on the dynamic changes of empty body mass, dry matter intake and milk production (Figures S13, S14, S15, S16, S17, S18, S19, S20, S21, S22, S23 and S24) [18, 19, 33–41]. [file 12711_2016_251_MOESM6_ESM.docx]

**Additional file 6 Calibration results and sensitivity analysis details**

**Calibration**

Figures S10, S11 and S12 illustrate our model calibration based on the outputs simulated by the GARUNS model [18, 19] and the compilation of literature data used as reference for a standard dairy cow.


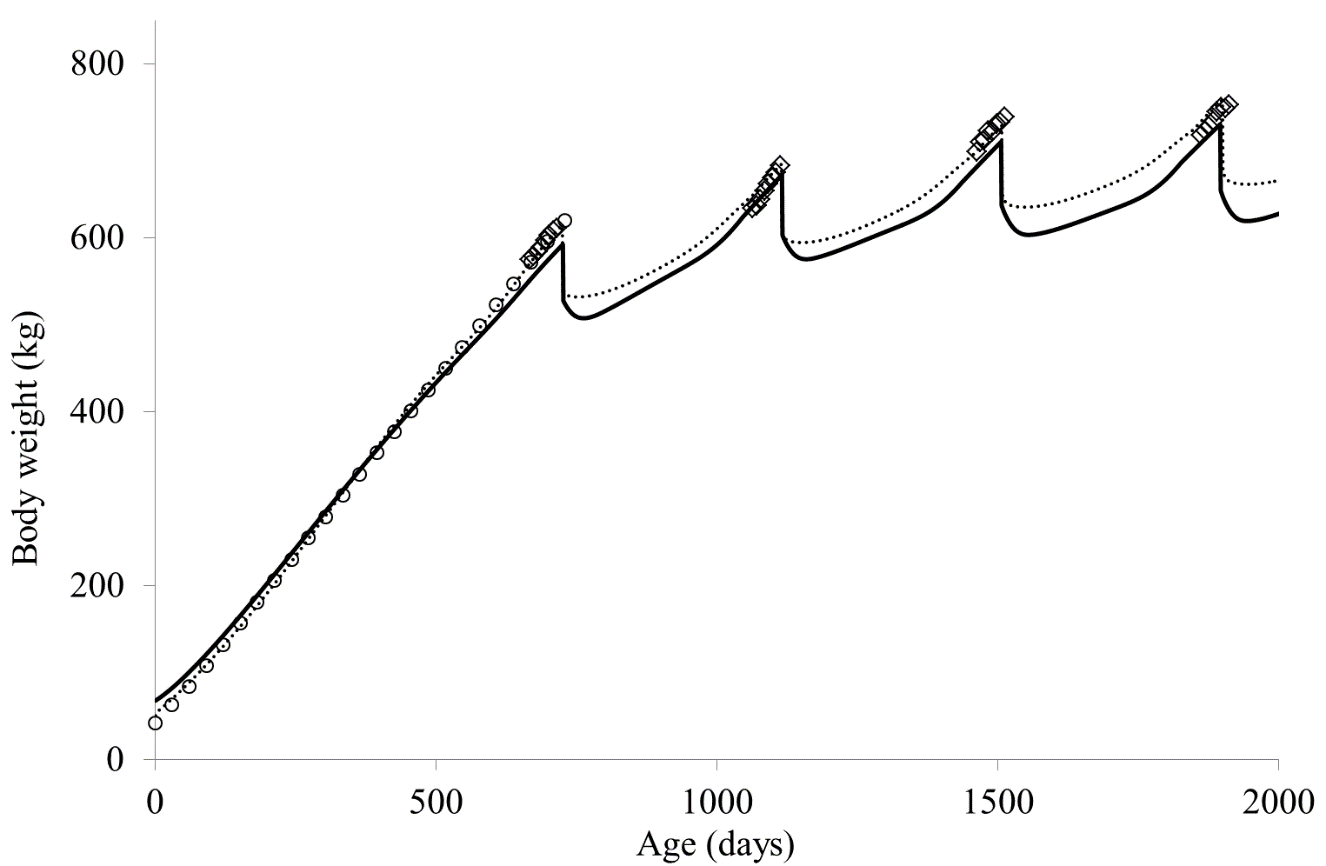


**Figure S10 Comparison of body mass simulated by the model (solid line), the GARUNS model (dotted line) and data from [33] (circles) and from [34] (diamonds).**


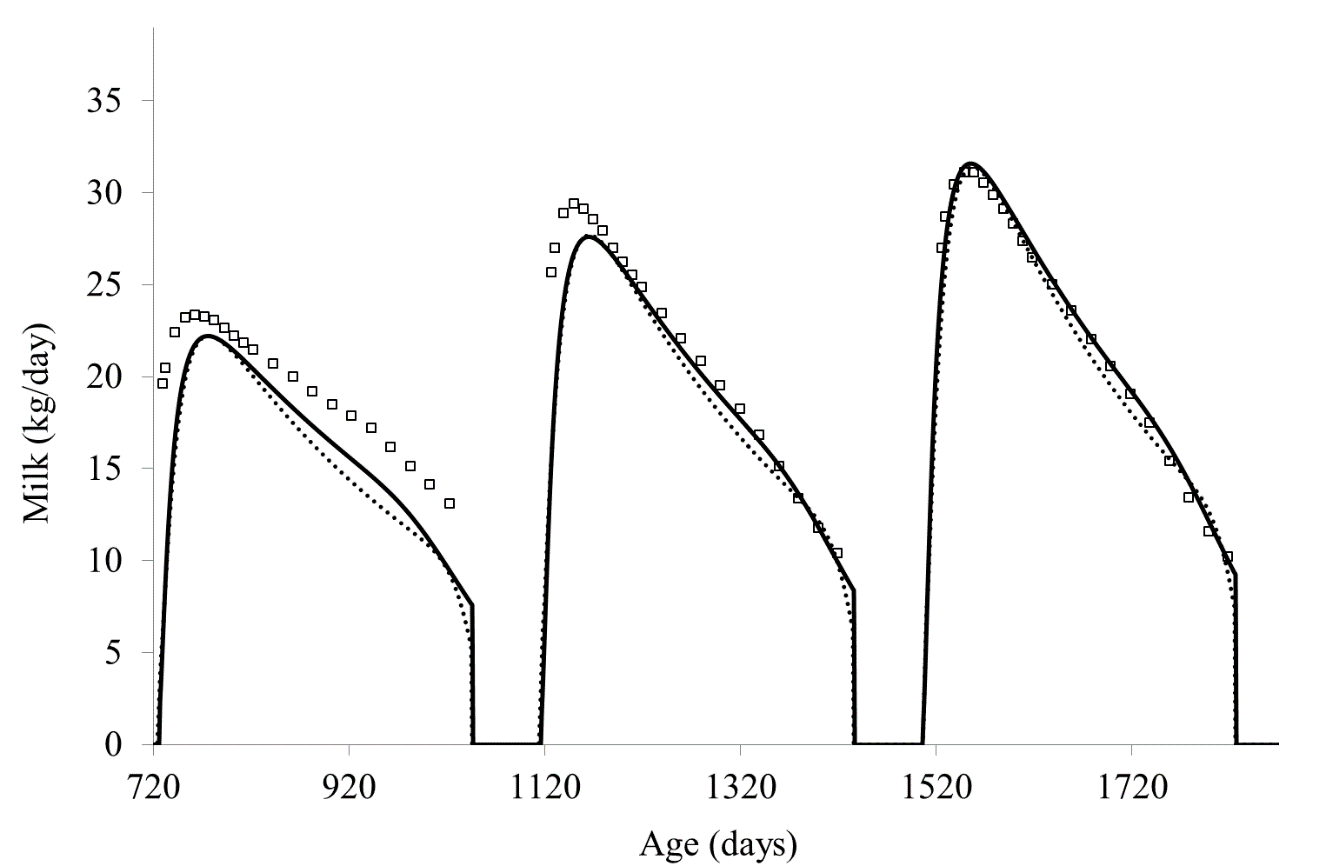


**Figure S11 Comparison of milk production simulated by the model (solid line), the GARUNS model (dotted line) and data from [35] (squares).**


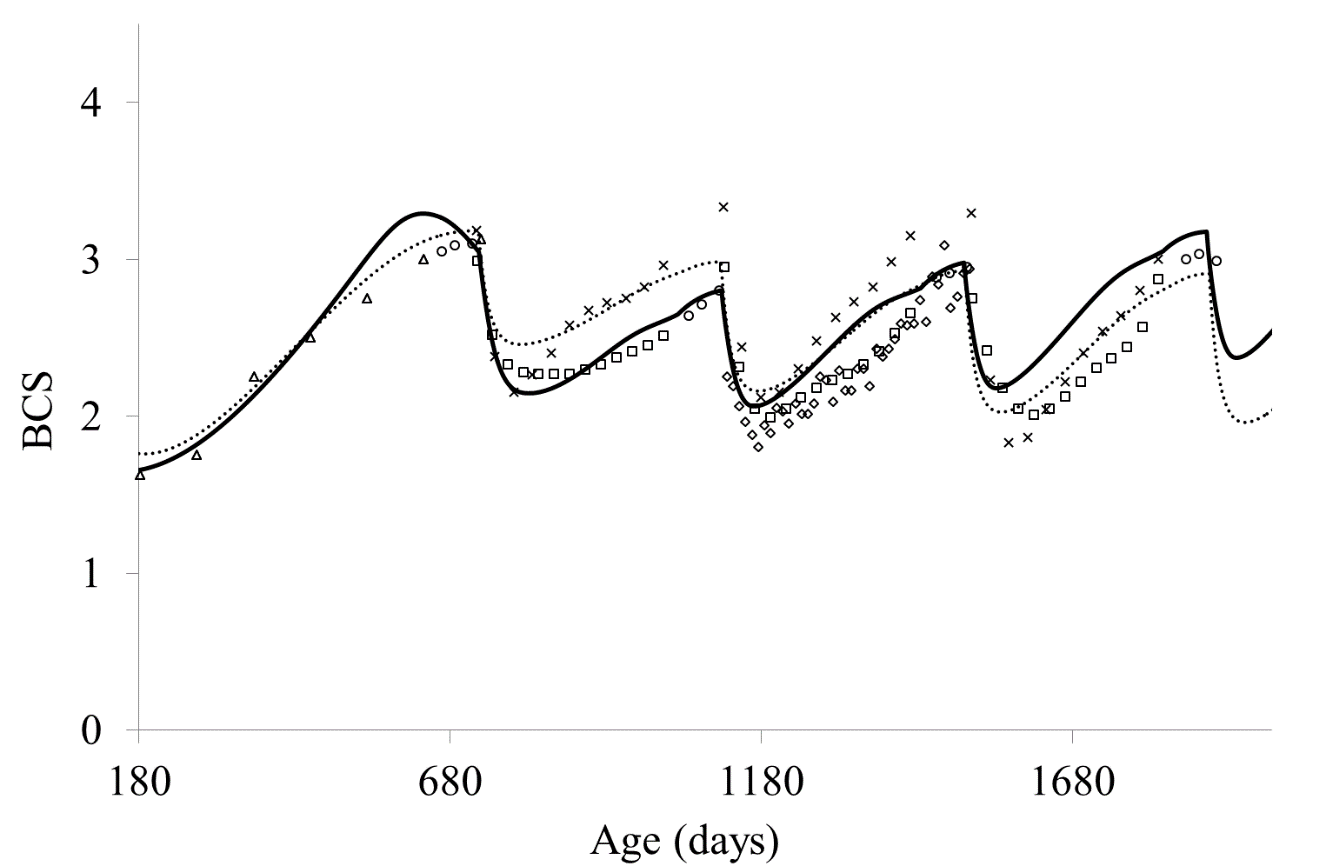


**Figure S12 Comparison of body condition score (BCS) simulated by the model (solid line), the GARUNS model (dotted line) and data from [33] (triangles), [34] (circles), [36] (diamonds), [37] (squares) and [38] (crosses).**

**Details description of the sensitivity analysis**

Model behaviour was explored through a global sensitivity analysis, aimed at evaluating how variation in model inputs, the four genetic-scaling parameters, affects FE. Two FE definitions were used, at lactation level and at the end of life. FE_Lac2 corresponds to the ratio between energy acquired and energy produced in milk, cumulated over the second lactation. FE_life corresponds to the same ratio, cumulated from birth to death. The four parameters ($G2S_{GEN}$,$Pc2S_{GEN}$, $AcqB_{GEN}$ and $AcqL_{GEN}$ ) were set at three different levels (L: low; M: medium and H: high) and combined in a complete factorial design. This led to a total of 81 simulations, with 20 replications to account for the stochastic processes of reproduction. The discretization of parameters into levels allowed a reasonable computation time while enabling the exploration of model behaviour in response to different combinations of values for genetic-scaling parameters. For each of the four genetic-scaling parameters, the medium level corresponded to the value determined in the calibration step [See Additional file 1 Table S11]. Values for low and high levels of parameters corresponded to equidistant deviations in percentage of the medium level. The % deviations were chosen to simulate trajectories of traits consistent with the range of trait values observed in existing data. By testing all the combinations of parameters values, different individual profiles of acquisition and allocation were simulated and the corresponding lifetime trajectories of traits were used to compute FE. The summary of values for genetic-scaling parameters used in sensitivity analysis is in Table S10. The deviations for the genetic-scaling parameter of basal acquisition were determined with data from [39]. We assumed that DM intake of heifers reflected basal acquisition level, apart from the effect of lactation. The mean value of Australian dairy heifers was 8.3 kg of DM/d, close to the medium level of the calibration step. High level of basal acquisition was set to 9.35 kg of DM/d and low level to 7.65 kg/d. These values corresponded to a 10% deviation from the medium level and were consistent with the range of variation observed by [39]. The same reference was used to determine deviations of the genetic-scaling parameter for lactation acquisition. We used the medium level from calibration step (11.5 kg of DM/d) and a 10% deviation to obtain the low level (10.35 kg of DM/d) and the high level (12.65 kg of DM/d) values for the parameter. The deviations for the genetic-scaling parameter for allocation to growth were determined based on body weight data from [40] and on levels determined for basal acquisition. Indeed, in the model, the structural body mass is determined by basal acquisition and allocation to growth [See Additional file 5]. For medium level values of basal acquisition and allocation to growth, the structural body mass was equal to 442.7 kg, corresponding approximately to 630 kg at first service for third parity and more. We had to take into account levels of basal acquisition in order to determine deviations for allocation to growth. We determined that with a 4 % deviation of the value for allocation to growth, combined to the 10 % deviation for basal acquisition previously mentioned the structural body mass varied from 383 to 508 kg and total body mass from 547 to 726 kg. This variation on body mass, around 14 % for high and low levels, was consistent with the variation reported by [34]. Finally, the deviations for the genetic-scaling parameter for allocation to lactation were determined in order to respect the culling criteria defined in the model. Culling occurred if the simulated cow didn’t become pregnant 200d after calving. Our aim was to explore the sensitivity of the model regarding two definitions of FE, one defined at second lactation level and one at lifespan level. To obtain different values for these two definitions of FE, we needed to simulate cow with at least three lactations, that is to say a cow not culled during the first three lactations. When value of allocation to lactation decreased, the level of body reserves increased and led to a decrease in conception probability. We determined that -5 % of the medium level value for lactation allocation was the lowest value possible to not simulate culled cow before the end of third lactation. As a result, we chose a +5 % symmetric deviation for the high level of lactation allocation.

**Table S4 Values of the four genetic-scaling parameters related to acquisition and allocation used in the factorial design for model sensitivity analysis**

|  |  | **Level** | | |
| --- | --- | --- | --- | --- |
|  |  | **Low** | **Medium** | **High** |
| Allocation to growth ($G2S_{GEN}$) | Value of parameter | 0.00276 | 0.00265 | 0.00254 |
|  | *Deviation from medium level (%)* | *4* |  | *-4* |
| Allocation to lactation ($Pc2S_{GEN}$) | Value of parameter | 0.00200 | 0.0016 | 0.00120 |
|  | *Deviation from medium level (%)* | *25* |  | *-25* |
| Basal acquisition ($AcqB_{GEN}$) | Value of parameter | 7.65000 | 8.5 | 9.35 |
|  | *Deviation from medium level (%)* | *-10* |  | *10* |
| Lactation acquisition ($AcqL_{GEN}$) | Value of parameter | 10.35000 | 11.5 | 12.65 |
|  | *Deviation from medium level (%)* | *-10* |  | *10* |

**Results of the sensitivity analysis**

Variance decomposition for the two definitions of FE of the results simulated with the factorial design used in the sensitivity analysis is in Table S5. The proportion of variance explained for an effect *X_i_* corresponded to the ratio between the conditional variance of *Y* given *X_i_* and the total variance of *Y*. The proportion of variance explained by principal effect of each genetic-scaling parameter corresponded to first-order sensitivity indices [41]. These indices reflect the relative importance of the different parameters in model outputs. For FE computed at the level of lifespan (FE_life), allocation to growth and lactation acquisition had the greater contribution to FE variation. Allocation to growth contributed to 20.8 % of FE variation. Lactation acquisition contributed to 26.2 % of FE variation. For FE computed at the level of second lactation (FE_lac2), allocation to lactation was the most important contributing factor (86.3 %). These results are consistent with the analysis of other variables presented in the main manuscript.

**Table S5 Variance decomposition of the two definition of feed efficiency used to evaluate model sensitivity to the four genetic-scaling parameters**

|  |  | FE_life | | FE_lac2 | |
| --- | --- | --- | --- | --- | --- |
| Effect | Degree of freedom | Sum of squares | Percentage of variance explained | Sum of squares | Percentage of variance explained |
| $S2Pc_{GEN}$ | 2 | 0.0932 | 6.0 | 1.1764 | 86.3 |
| $G2S_{GEN}$ | 2 | 0.3223 | 20.8 | 0.0033 | 0.2 |
| $AcqB_{GEN}$ | 2 | 0.0275 | 1.8 | 0.0003 | 0.0 |
| $AcqL_{GEN}$ | 2 | 0.4051 | 26.2 | 0.0005 | 0.0 |
| $Pc2S_{GEN} \cdot G2S_{GEN}$ | 4 | 0.1129 | 7.3 | 0.0015 | 0.1 |
| $Pc2S_{GEN}\cdot AcqB_{GEN}$ | 4 | 0.0213 | 1.4 | 0.0007 | 0.1 |
| $G2S_{GEN}\cdot AcqB_{GEN}$ | 4 | 0.0207 | 1.3 | 0.0018 | 0.1 |
| $Pc2S_{GEN}\cdot AcqL_{GEN}$ | 4 | 0.1239 | 8.0 | 0.001 | 0.1 |
| $G2S_{GEN}\cdot AcqL_{GEN}$ | 4 | 0.0184 | 1.2 | 0.001 | 0.1 |
| $AcqB_{GEN}\cdot AcqL_{GEN}$ | 4 | 0.0087 | 0.6 | 0.0007 | 0.1 |
| $Pc2S_{GEN}\cdot G2S_{GEN}\cdot AcqB_{GEN}$ | 8 | 0.0022 | 0.1 | 0.0036 | 0.3 |
| $Pc2S_{GEN}\cdot G2S_{GEN}\cdot AcqL_{GEN}$ | 8 | 0.0332 | 2.1 | 0.0053 | 0.4 |
| $Pc2S_{GEN}\cdot AcqB_{GEN}\cdot AcqL_{GEN}$ | 8 | 0.0043 | 0.3 | 0.0013 | 0.1 |
| $G2S_{GEN}\cdot AcqB_{GEN}\cdot AcqL_{GEN}$ | 8 | 0.0065 | 0.4 | 0.0024 | 0.2 |
| $Pc2S_{GEN}\cdot G2S_{GEN}\cdot AcqB_{GEN}\cdot AcqL_{GEN}$ | 16 | 0.0208 | 1.3 | 0.0235 | 1.7 |
| $Residuals$ | 1539 | 0.325 | 21.0 | 0.1393 | 10.2 |

FE_life: ratio between cumulative energy for milk production and cumulative energy acquired, from birth to death. FE_lac2: ratio between cumulative energy for milk production and cumulative energy acquired, from second parturition to second drying-off. The sensitivity analysis was based on a complete factorial design combining 3 levels (L: low; M: medium and H: high) of the 4 genetic-scaling parameters driving allocation to growth ($G2S_{GEN}$), allocation to lactation ($Pc2S_{GEN}$), basal acquisition ($AcqB_{GEN}$) and lactation acquisition ($AcqL_{GEN}$).

Figures S5, S6, S7, S8, S9, S10, S11, S12, S13, S14, S15, and S16 illustrate the effects of parameters related to allocation to growth ($G2S_{GEN}$), allocation to lactation ($Pc2S_{GEN}$), basal acquisition ($AcqB_{GEN}$) and lactation acquisition ($AcqL_{GEN}$) tested in the factorial design on body mass components, milk production and dry matter intake lifetime trajectories. Individual selected for illustrative purpose were the closest to median values for lactation number, structural mass, life efficiency, lactation 2 efficiency, lifetime DMI and lifetime milk production.


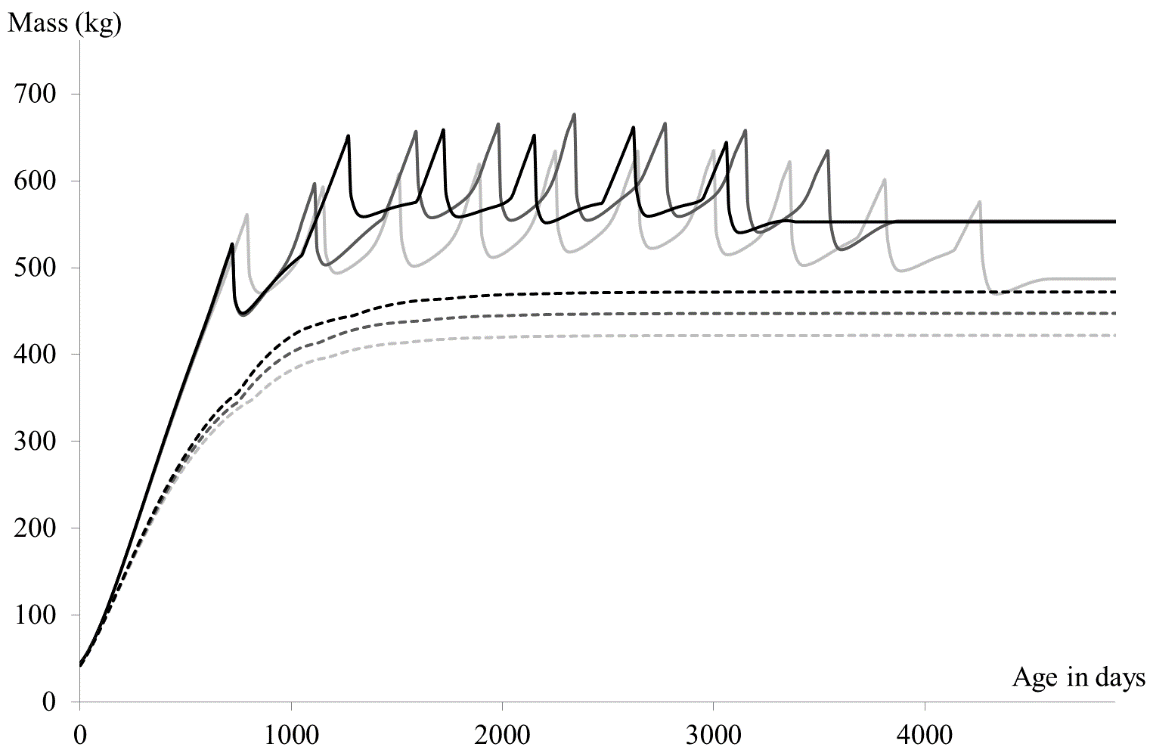


**Figure S13 Empty body mass trajectories (solid lines) and structural mass trajectories (dotted lines) for individuals with L (light grey), M (dark grey) and H (black) levels of allocation to growth.**


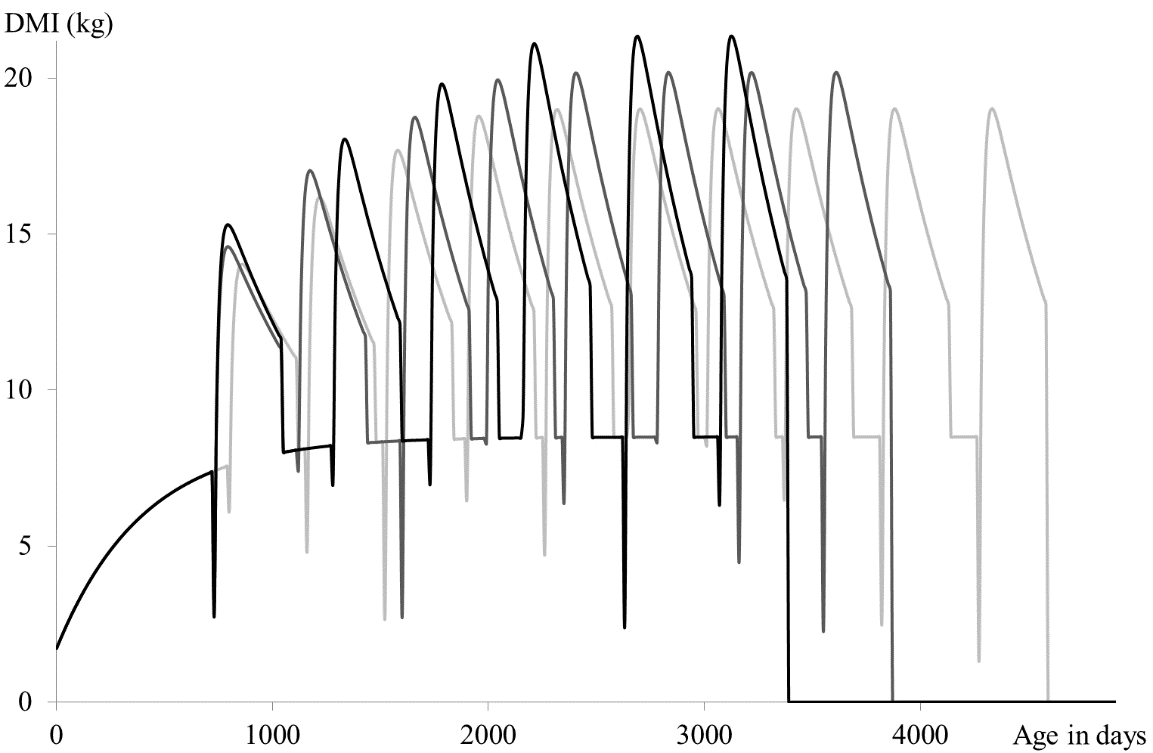


**Figure S14 Dry matter intake trajectories (DMI) for individuals with L (light grey), M (dark grey) and H (black) levels of allocation to growth.**


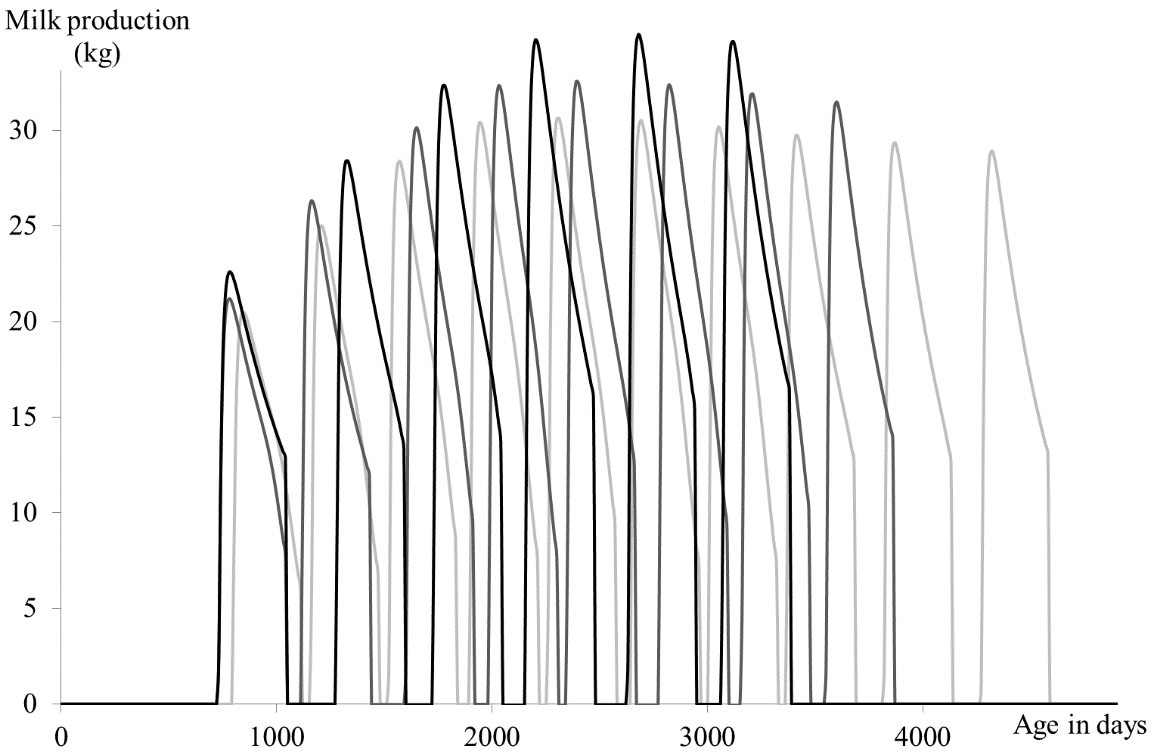


**Figure S15 Milk production trajectories (solid lines) and structural mass trajectories for individuals with L (light grey), M (dark grey) and H (black) levels of allocation to growth.**


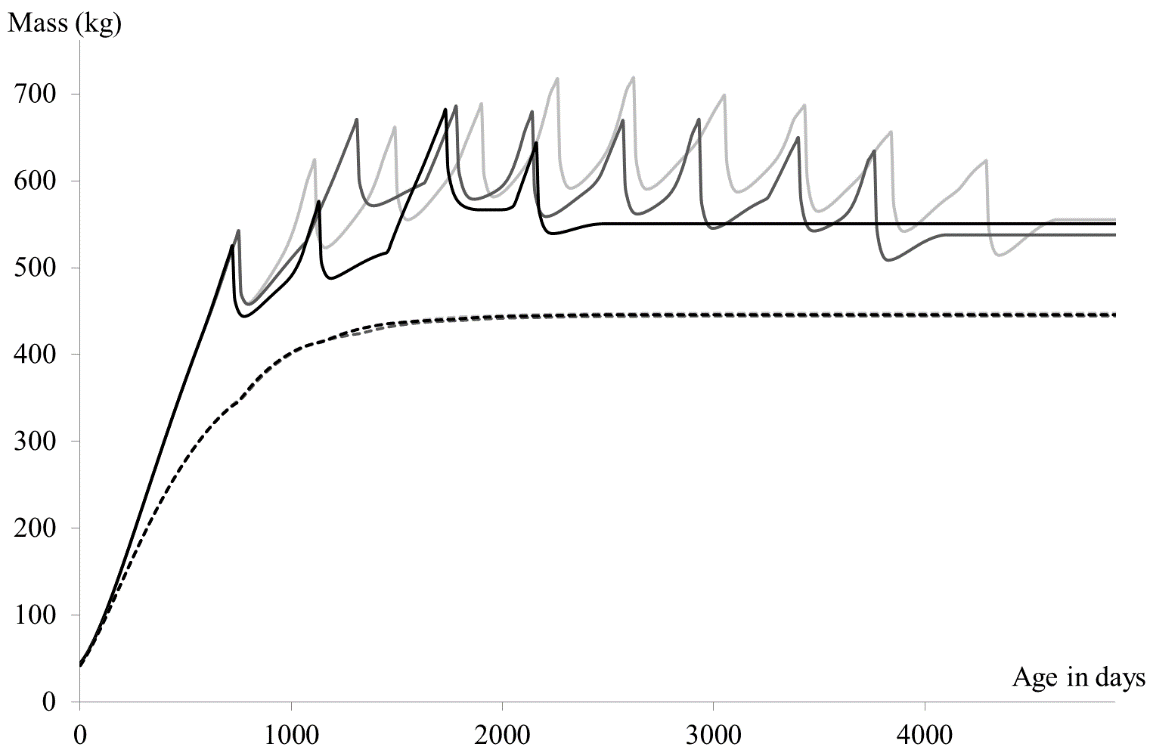


**Figure S16 Empty body mass trajectories (solid lines) and structural mass trajectories (dotted lines) for individuals with L (light grey), M (dark grey) and H (black) levels of allocation to lactation.**


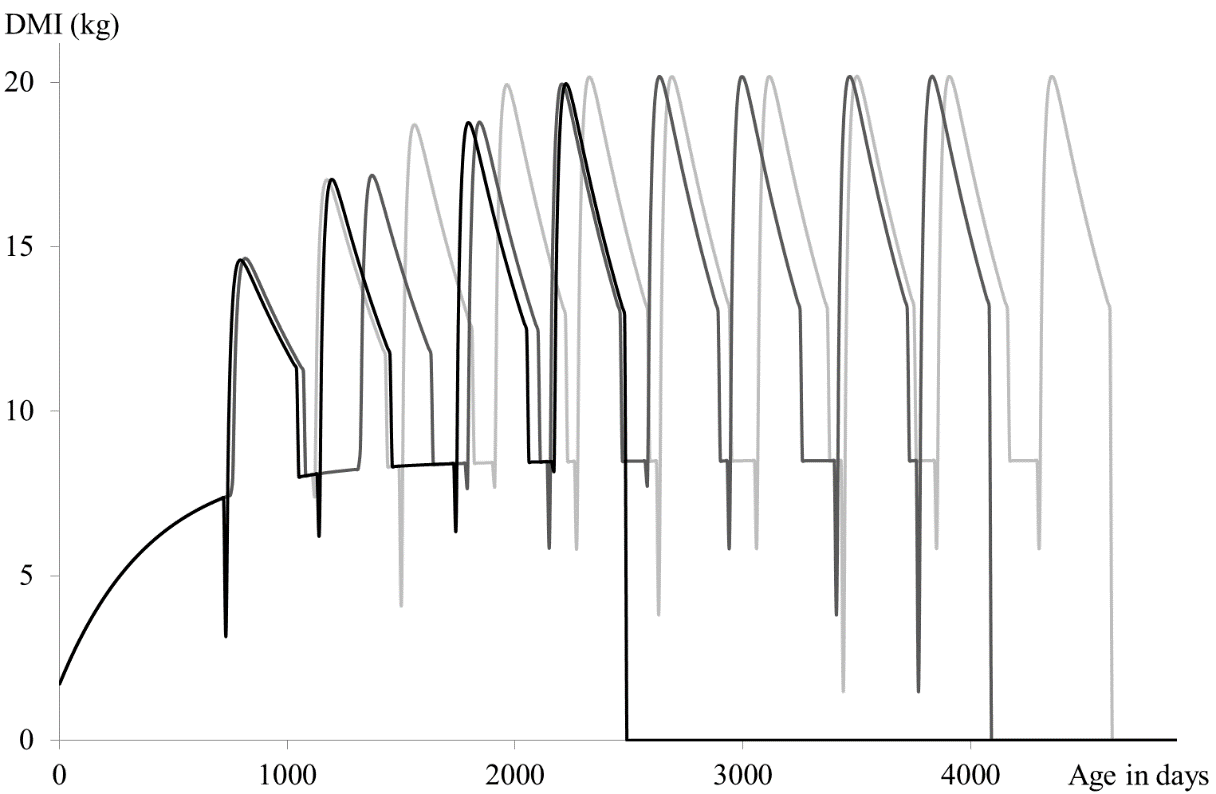


**Figure S17 Dry matter intake (DMI) trajectories for individuals with L (light grey), M (dark grey) and H (black) levels of allocation to lactation.**


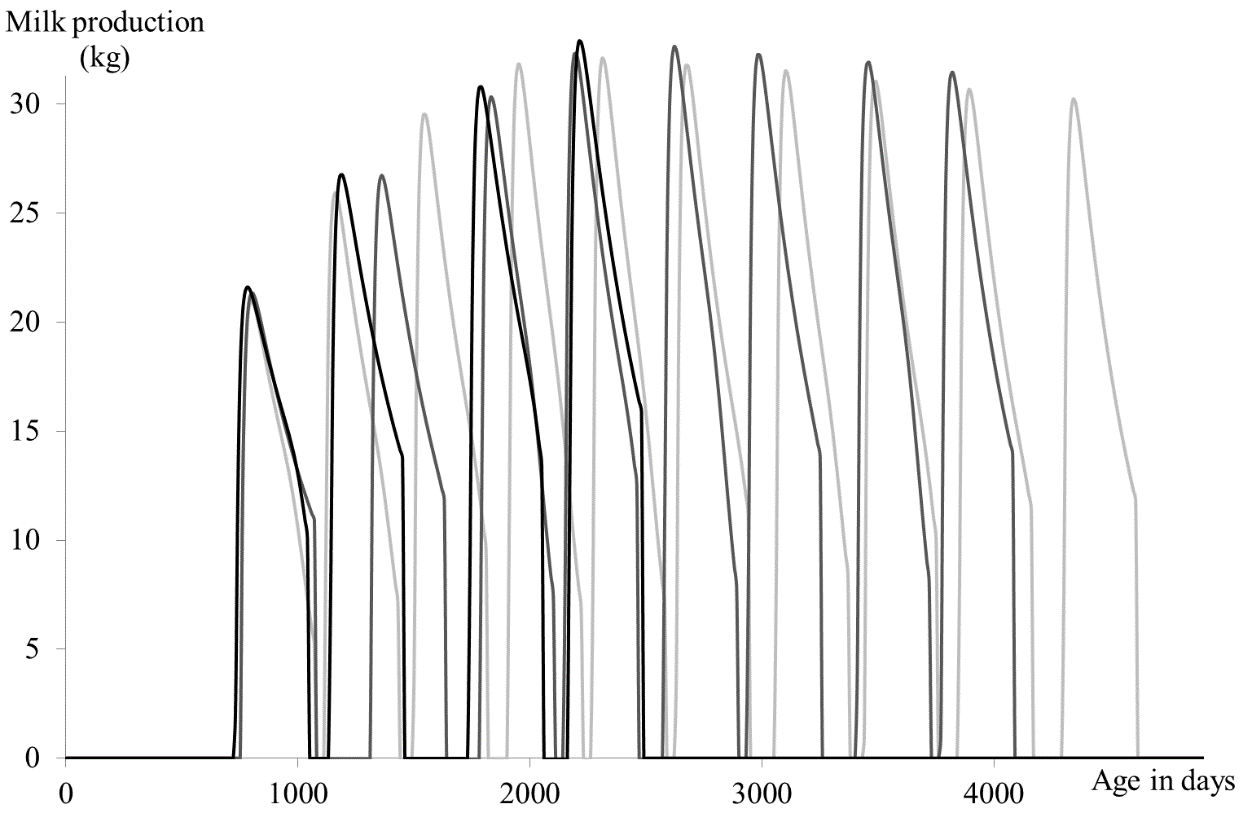


**Figure S18 Milk production trajectories (solid lines) and structural mass trajectories for individuals with L (light grey), M (dark grey) and H (black) levels of allocation to lactation.**


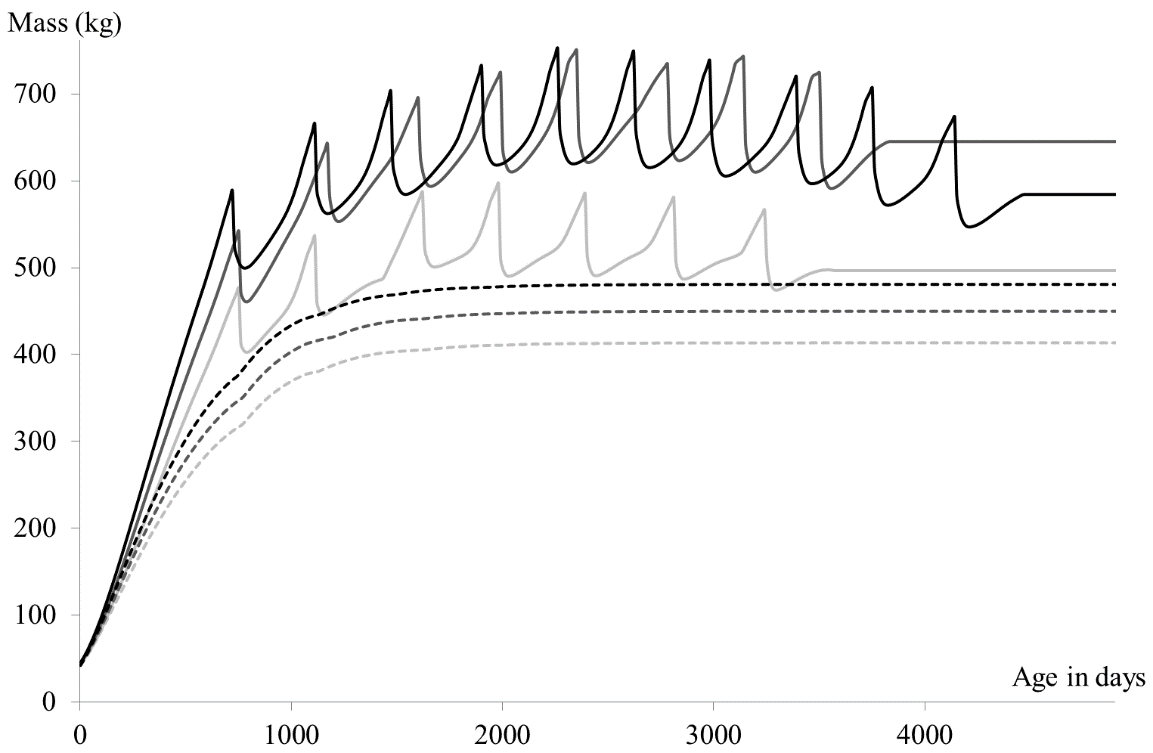


**Figure S19 Empty body mass trajectories (solid lines) and structural mass trajectories (dotted lines) for individuals with L (light grey), M (dark grey) and H (black) levels of basal acquisition.**


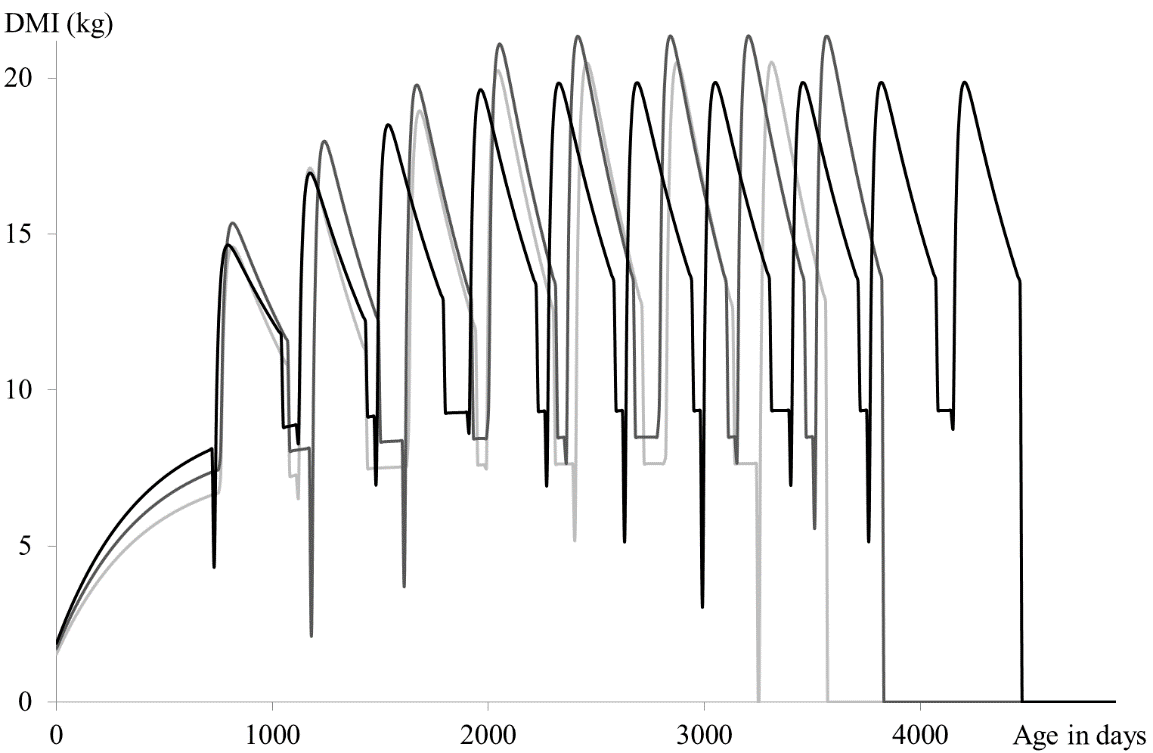


**Figure S20 Dry matter intake (DMI) trajectories for individuals with L (light grey), M (dark grey) and H (black) levels of basal acquisition.**


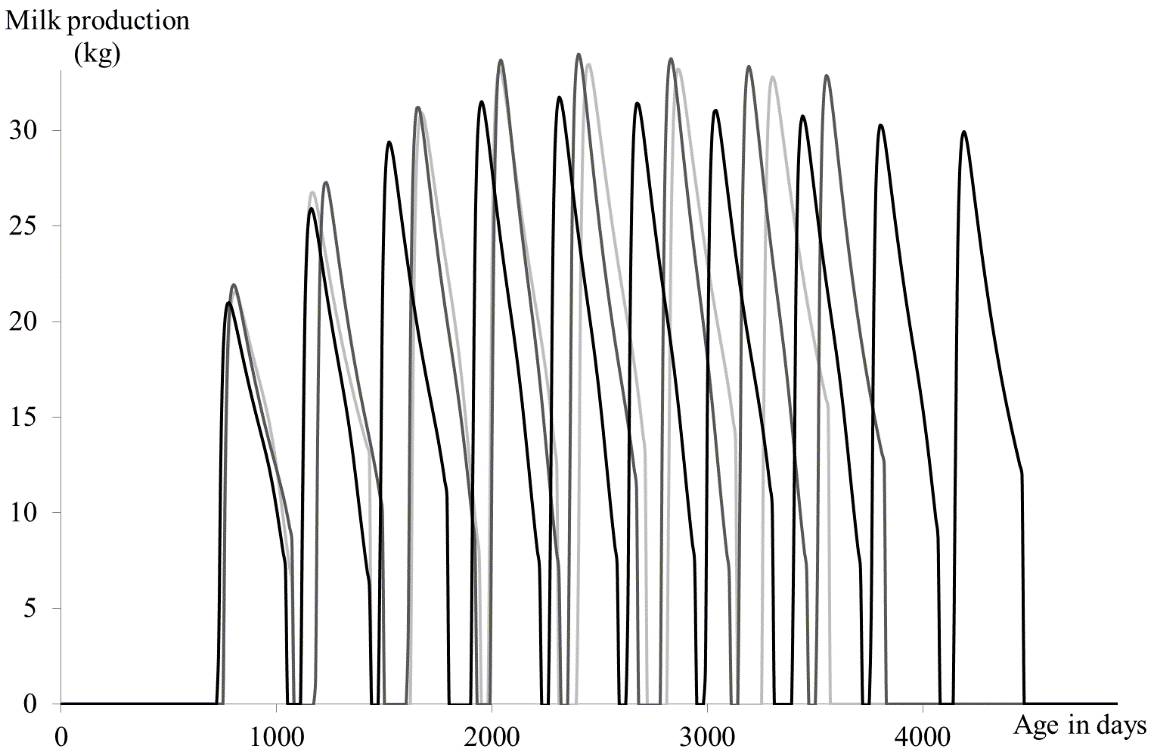


**Figure S21 Milk production trajectories (solid lines) and structural mass trajectories for individuals with L (light grey), M (dark grey) and H (black) levels of basal acquisition.**


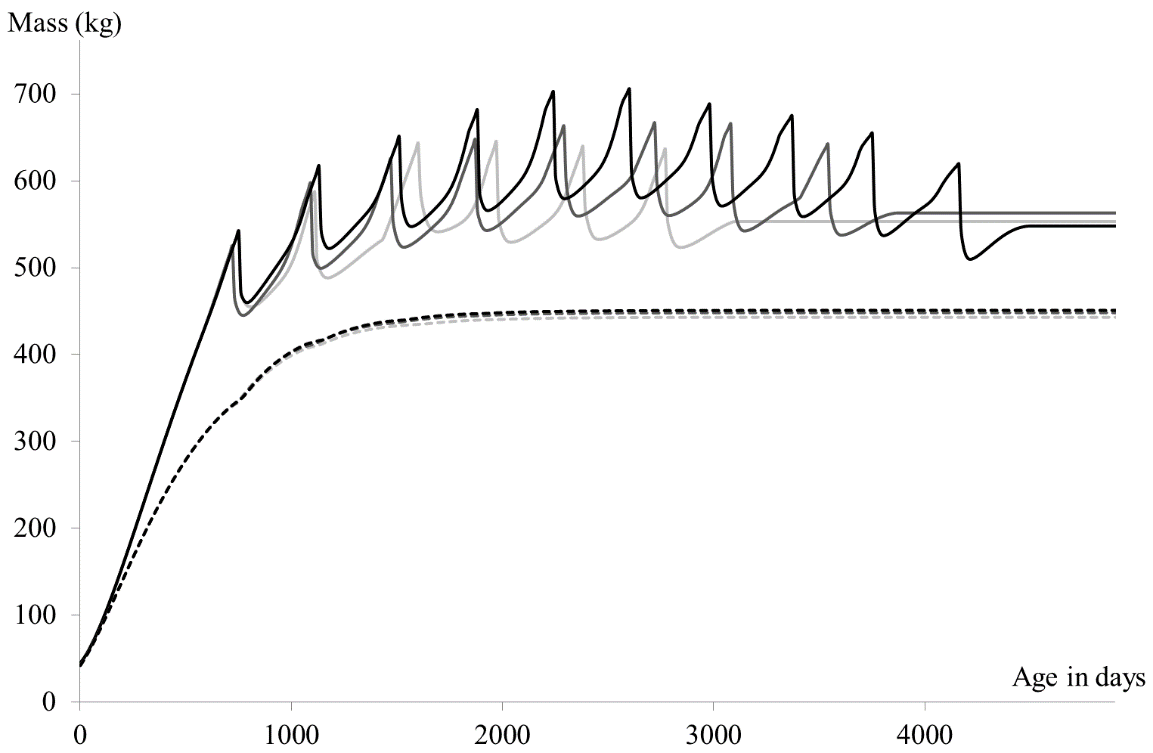


**Figure S22 Empty body mass trajectories (solid lines) and structural mass trajectories (dotted lines) for individuals with L (light grey), M (dark grey) and H (black) levels of lactation acquisition.**


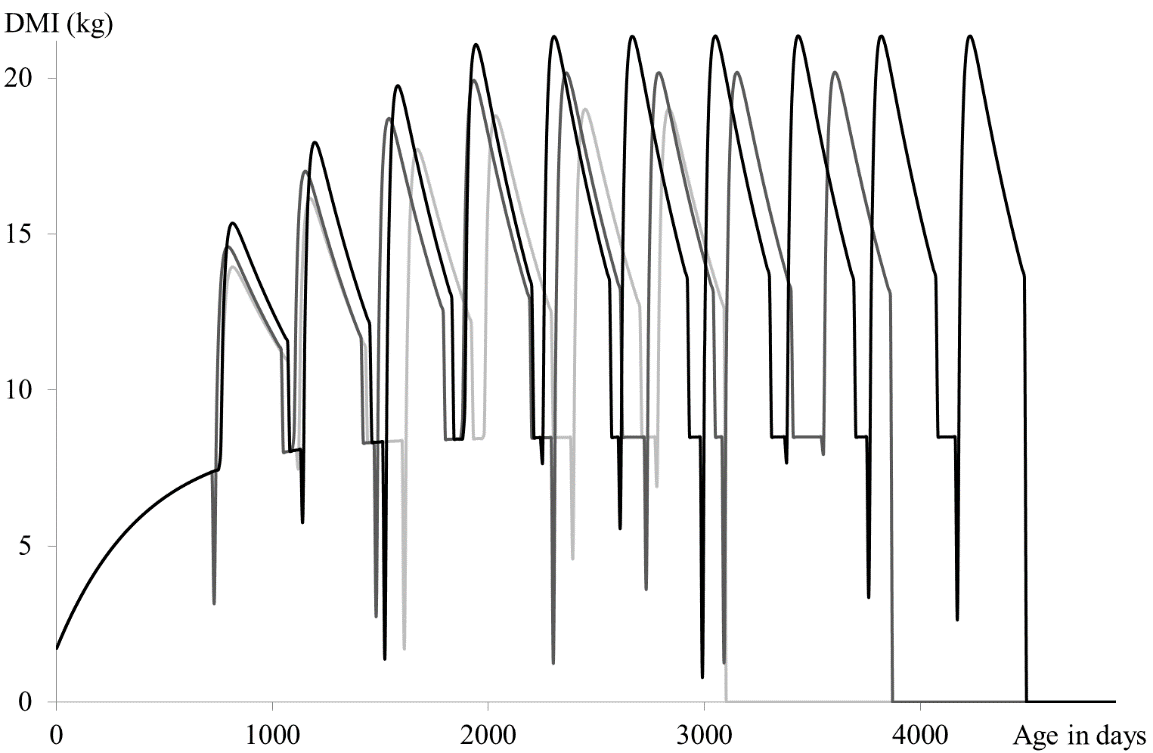


**Figure S23 Dry matter intake (DMI) trajectories for individuals with L (light grey), M (dark grey) and H (black) levels of lactation acquisition.**


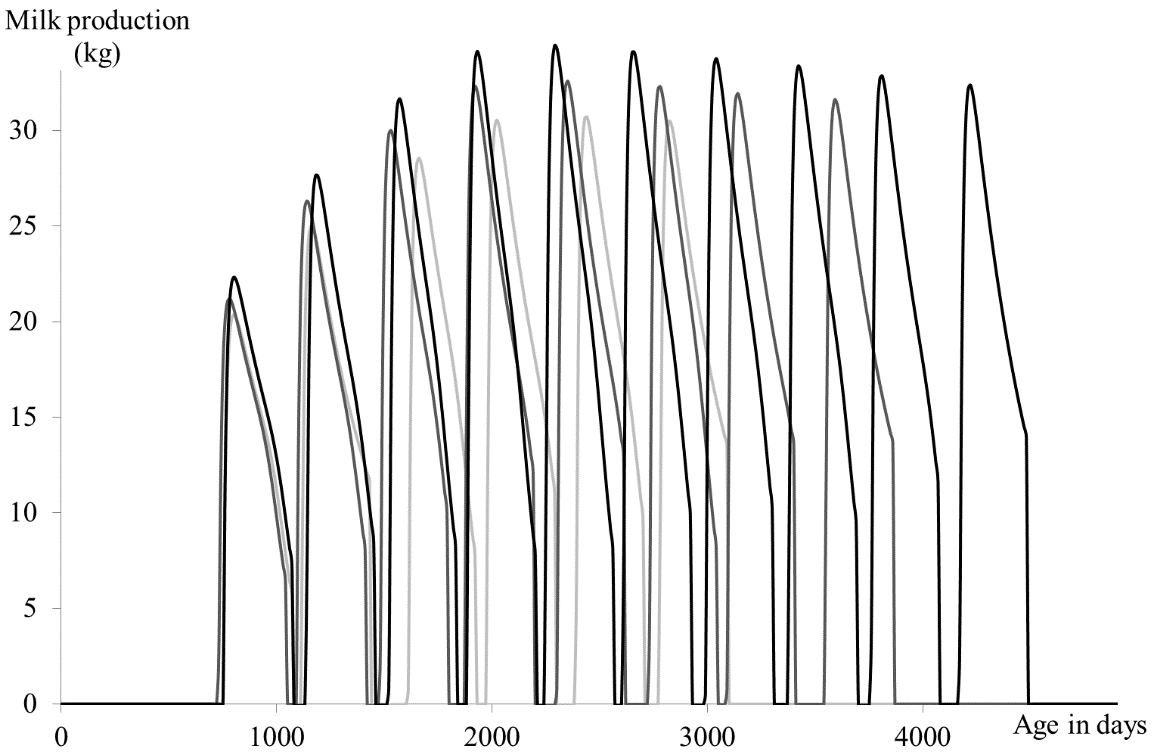


**Figure S24 Milk production trajectories (solid lines) and structural mass trajectories for individuals with L (light grey), M (dark grey) and H (black) levels of lactation acquisition.**
